# *Pelargonium zonale* as a Source of Active Catalysts for the Oxidation of Geraniol with Oxygen

**DOI:** 10.3390/ma19163468

**Published:** 2026-08-17

**Authors:** Sylwia Gajewska, Joanna Siemak, Agnieszka Wróblewska, Luis A. Gallego-Villada, Beata Michalkiewicz

**Affiliations:** 1 Faculty of Chemical Technology and Engineering, Department of Catalytic and Sorbent Materials Engineering, West Pomeranian University of Technology in Szczecin, Piastów Ave. 42, 71-065 Szczecin, Poland; gs54727@zut.edu.pl (S.G.); joanna.siemak@zut.edu.pl (J.S.); 2 Laboratory of Industrial Chemistry and Reaction Engineering, Johan Gadolin Process Chemistry Centre, Åbo Akademi University, Henriksgatan 2, 20500 Turku, Finland; luis.gallegovillada@abo.fi; 3 West Pomeranian University of Technology in Szczecin, Faculty of Chemical Technology and Engineering, Center for Advanced Materials and Manufacturing Process Engineering MATPRO, al. Piastów 42, 71-065 Szczecin, Poland

**Keywords:** activated carbons from *Pelargonium zonale*, dried powder from *Pelargonium zonale*, geraniol oxidation, 2,3-epoxygeraniol, 2,3-epoxycitral, citral

## Abstract

The sustainable utilization of post-consumer plant biomass as a precursor for functional carbon materials has attracted increasing attention as an environmentally friendly approach to heterogeneous catalyst development. In this study, the aerial parts of *Pelargonium zonale* were converted into activated carbons by chemical activation with KOH at carbonization temperatures of 750, 800, and 850 °C. The obtained materials were comprehensively characterized using N_2_ adsorption–desorption, XRD, FTIR, UV–Vis spectroscopy, XRF, and SEM. Increasing the carbonization temperature promoted pore development, resulting in activated carbons with a maximum specific surface area of 1578 m^2^ g^−1^ and a total pore volume of 0.751 cm^3^ g^−1^**.** The catalytic performance of the obtained materials was evaluated in the oxidation of geraniol. No direct correlation between the textural properties of the activated carbons and their catalytic performance was observed, suggesting that catalytic behaviour results from the combined effect of several physicochemical properties. Optimization of the reaction conditions demonstrated that catalytic performance depended on the combined influence of reaction temperature, catalyst amount, reaction time, and catalyst properties. Under the optimized reaction conditions (110 °C, 0.5 wt% catalyst, and 360 min), the highest geraniol conversion (37 mol%) was achieved over CP_KOH_850. Kinetic modelling confirmed that geraniol oxidation proceeds through a complex network of parallel and consecutive reactions leading to the formation of citral, 2,3-epoxygeraniol, 2,3-epoxycitral, and other oxidation products. These findings demonstrate that waste *Pelargonium zonale* biomass is a promising renewable precursor for the preparation of functional carbon materials for heterogeneous catalytic applications.

## 1. Introduction

Characterized by a fresh and intense scent, geraniol, an unsaturated terpene alcohol, occurs naturally in the essential oils of plants such as lime, ginger, and lemon [1]. Its principal natural source is Pelargonium, from which it is most commonly extracted. Due to its pleasant aroma, geraniol is widely employed in the formulation of diverse fragrance blends [2]. A pure natural compound, it exhibits diverse pharmacological activities, including anticancer, anti-inflammatory, antioxidant, antifungal, antidiabetic, and antinociceptive effects, primarily through regulation of protein expression, suggesting its potential as a novel therapeutic agent [3]. However, it can also be used to produce other valuable compounds. Terpenes such as geraniol are naturally occurring substances that readily undergo conversion into high-value derivatives, which find broad application in the industrial synthesis of fragrances, flavoring agents, and therapeutic compounds [4]. Geraniol undergoes various transformations when exposed to porous catalytic materials, mainly leading to the production of 2,3-epoxygeraniol, 2,3-epoxycitral and citral [1]. For example, the last of these exhibits notable anti-inflammatory, antimicrobial, and antioxidant properties, making it a promising candidate for the treatment of inflammatory and neurodegenerative diseases [5]. In the field of catalytic reactions, the development and discovery of novel, highly efficient catalysts are actively being pursued.

Activated carbon can be a representative example of a porous material that not only serves as an effective support for various catalysts but also exhibits intrinsic catalytic activity due to its high surface area, structural heterogeneity, and surface functional groups [6,7]. Owing to its adsorptive properties, it is employed in diverse industrial branches, such as the food and pharmaceutical industries, as well as in wastewater treatment processes or energy storage [8]. The physicochemical properties of activated carbons are strongly influenced by the biomass precursor, carbonization conditions, and activation strategy, which together determine the development of pore structure, surface chemistry, and catalytic performance [9,10]. It was demonstrated that phosphoric acid-activated carbon (ACC) serves as a highly effective catalyst for converting glucose into phenol-rich bio-oils, achieving up to 100% phenol selectivity under optimal conditions, further noting that acid activation enhances phenol selectivity while alkali treatments improve deoxygenation pathways [11]. The catalytic activity of carbon-based materials has been well documented, for instance, in the oxidation of α-pinene to valuable oxygenated derivatives such as α-pinene oxide, verbenol, and verbenone [7]. In addition to conventional activation methods, advanced synthesis strategies, including hydrothermal carbonization and template-assisted synthesis, have recently been investigated to tailor the pore architecture and surface properties of porous carbon materials for adsorption and catalytic applications [12,13]. Various types of biomasses can be utilized as precursors for the synthesis of activated carbons, for instance, lignocellulosic materials derived from agricultural by-products such as banana and citrus peels, as well as forest residues like pine cones and also certain plant species, such as the common polypody (*Polypodium vulgare*) [7,14,15,16]. Lignocellulosic biomass has attracted particular attention because of its renewable nature, high carbon content, and ability to produce activated carbons with well-developed porous structures after physical or chemical activation [12,13]. In light of this, this work is focused on *Pelargonium zonale*. Similar to numerous other medicinal plants, species of the genus Pelargonium possess a diverse spectrum of bioactive compounds that confer multiple therapeutic properties. Recent studies have demonstrated that Pelargonium extracts exhibit pronounced antifungal and anticancer activities, underscoring their potential as valuable sources of natural compounds for biomedical research [17]. Moreover, Geraniums are globally valued for their remarkable ornamental qualities and wide range of horticultural applications [18]. Throughout its annual life cycle, this plant undergoes a series of distinct developmental stages characteristic of its natural growth and senescence processes. During the autumn and winter seasons, the aerial parts such as stems and leaves undergo desiccation. After the growing season, the above-ground biomass becomes a readily available post-consumer plant residue that can be sustainably valorized as a renewable precursor for the preparation of activated carbons, thereby supporting waste biomass utilization within the framework of a circular economy. This observation prompted the idea of exploring effective methods for utilizing these above-ground parts. One potential approach to their efficient application involves their use as catalysts, either in the form of simply dried and ground material or as activated carbons prepared from the same plant biomass.

The aim of this study was the preparation of activated carbons from the plant *Pelargonium zonale* using KOH as an activating agent, at carbonization temperatures of 750, 800, and 850 °C in a nitrogen atmosphere. The powder *Pelargonium zonale,* as well as the obtained activated carbons, were subjected to the oxidation process. Plant material was used after the end of the vegetation period. The oxidation process was carried out in three stages, with a separate parameter (temperature, catalyst amount and reaction time) tested in each stage. During the oxidation of geraniol on carbon-based catalysts and powder of *Pelargonium zonale*, three main products were obtained: citral, 2,3-epoxygeraniol, and 2,3-epoxycitral. Kinetic studies were performed for two catalysts: activated carbon from *Pelargonium zonale* carbonized at 850 °C and powder of *Pelargonium zonale*.

## 2. Materials and Methods

### 2.1. Preparation of Plant Material for Research

During preparation of *Pelargonium zonale* powder, leaves and stems of *Pelargonium zonale* were air-dried for six months in a well-ventilated room at a temperature of 15 °C. The dried material was then ground using a laboratory grinder and subsequently dried in a laboratory dryer at 50 °C for 24 h. After drying, the material was ground again with a laboratory grinder to obtain a fine powder and subjected to an additional drying step under the same conditions. The resulting *Pelargonium zonale* powder was utilized as a catalyst in the geraniol oxidation reaction.

**Figure 1:**
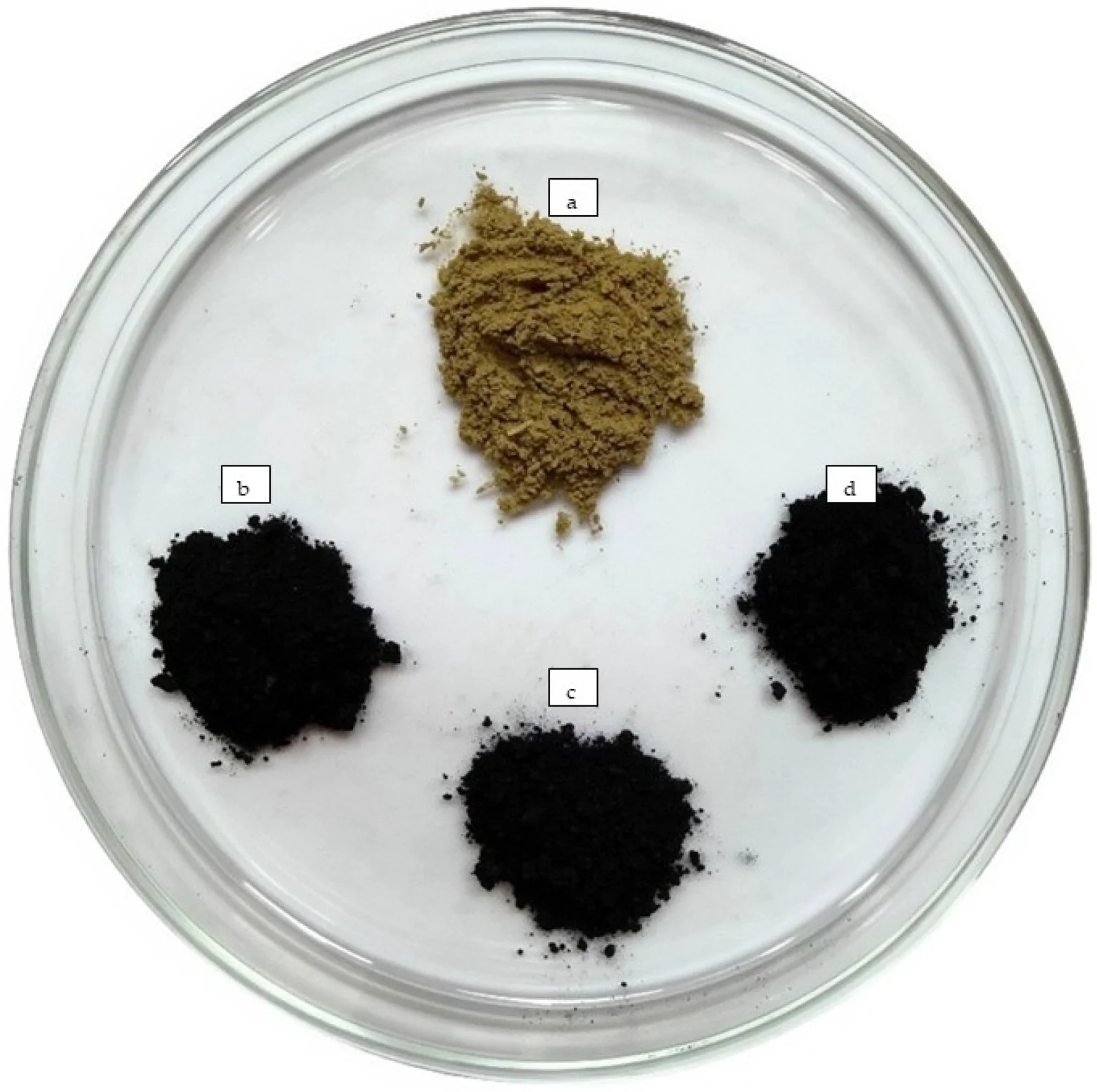
The image shows: a—P_POWDER, b—CP_KOH_750, c—CP_KOH_800, and d—CP_KOH_850.

For activated carbons the preparation was as follows. The obtained powder from *Pelargonium zonale* was impregnated with a saturated potassium hydroxide solution (KOH pure p.a.) (Stanlab, Lublin, Poland) in a 1:1 ratio at room temperature for 3 h and subsequently placed in a laboratory dryer for 18 h. Carbonisation was conducted in a tubular furnace at 750 °C, 800 °C and 850 °C under a nitrogen atmosphere for 1 h. Following carbonization, the sample was washed with 1 M hydrochloric acid (HCl pure p.a.) (Chempur, Piekary Śląskie, Poland) until an acidic pH was achieved and further rinsed with distilled water until a neutral pH was obtained. Finally, the material was dried in a laboratory dryer at 120 °C for 18 h. Figure 1 presents the catalysts used in the oxidation of geraniol with oxygen: a—P_POWDER, b—CP_KOH_750, c—CP_KOH_800, and d—CP_KOH_850.

### 2.2. Instrumental Methods Used for the Analysis of the Obtained Catalysts (P_POWDER, CP_KOH_750, CP_KOH_800, and CP_KOH_850)

Materials such as P_POWDER and CP_KOH_750, CP_KOH_800, and CP_KOH_850 were subjected to instrumental analyses to examine the composition, properties, and characteristics of the catalysts. Subsequently, catalytic tests were carried out. The specific surface area (SSA), total pore volume (Vtot), micropore volume (Vmicro), pore size distribution, and adsorption isotherms were determined using nitrogen (N_2_) adsorption measurements. These analyses were carried out with an ASAP 2460 Surface Area and Pore Size Analyzer (Micromeritics, Norcross, GA, USA, 2018). The pore size distributions were calculated from the nitrogen adsorption isotherms using the Density Functional Theory (DFT) method implemented in the Micromeritics MicroActive 3.01 software. The DFT model is based on statistical mechanics and enables the determination of pore size distributions by fitting theoretical adsorption isotherms to the experimental data. DFT method was calculated from the adsorption branch of the nitrogen adsorption isotherm. The total pore volume was determined from the amount of nitrogen adsorbed as the relative pressure approached 1. X-ray diffraction (XRD) patterns were recorded using an X’Pert PRO diffractometer (Panalytical, Almelo, The Netherlands, 2012) equipped with a copper radiation source. All analyses were carried out using a Elemental composition was determined using a Bruker S8 TIGER wavelength-dispersive X-ray fluorescence (WD-XRF) spectrometer (Karlsruhe, Germany) equipped with a Rh-anode X-ray tube, a flow proportional detector for light elements, and a scintillation detector for medium and heavy elements. Analyses were performed directly on loose powder samples without pelletization or fusion under a helium atmosphere. The infrared spectrum (FTIR) in the wavenumber range of 400–4000 cm^−1^ was obtained using a Thermo Electron Nicolet 380 spectrometer (Malente, Germany). UV–Vis diffuse reflectance spectra (DRS) of the powdered samples were recorded in the wavelength range of 190–900 nm using a Jasco V-650 spectrophotometer (JASCO Corporation, Tokyo, Japan) equipped with a diffuse reflectance accessory. Field emission scanning electron microscopy images were obtained using a SU8020 Ultra-High Resolution FE-SEM (Hitachi Ltd., Tokyo, Japan, 2012) operated at 15 kV.

### 2.3. Oxidation of Geraniol on Dried Powder of Pelargonium zonale and Activated Carbons from Pelargonium zonale

The oxidation of geraniol was carried out in an apparatus consisting of a glass three-neck reactor with a volume of 25 cm^3^, an oil bath, a magnetic stirrer with a heating function, an oxygen cylinder with a purity of 99.99% (Messer, Szczecin, Poland), a flow meter supplying oxygen to the reactor at a rate of 40 mL/min, and a reflux condenser. For the catalytic studies on the effect of temperature and catalyst amount, 10 g of geraniol (97%, Sigma Aldrich, Poznań, Poland) was weighed into the glass reactor using an analytical balance. For the series of experiments investigating the influence of reaction time, 20 g of geraniol was used. Subsequently, the appropriate amount of catalyst (P_POWDER or CP_KOH_750, CP_KOH_800, CP_KOH_850) was added to the reactor. The reactor was immersed in the oil bath using a laboratory clamp. Next, a glass gas distributor and a reflux condenser were placed in the reactor. The reaction mixture was stirred at 500 rpm. This stirring speed, combined with the use of catalyst particles smaller than 100 µm, ensured efficient mixing and minimized both external and internal mass transfer limitations under the applied reaction conditions. A series of catalytic tests were performed to examine the effects of temperature (80–110 °C), catalyst amount (0.5–5 wt%), and reaction time (15–360 min). Each catalytic experiment was performed independently using a fresh catalyst sample.

Post-reaction mixtures were sampled using pipettes, with 1 mL transferred into Eppendorf tubes and then centrifuged in a laboratory centrifuge. The samples were subsequently diluted with acetone (99.5%, Pol-Aura, Morąg, Poland) at a ratio of 1:4. The composition of each post-reaction mixture was determined by GC analysis performed in triplicate. The analysis was conducted using gas chromatography on a FOCUS instrument (Thermo Fisher Scientific, Waltham, MA, USA) equipped with an FID detector. Separation was achieved with a ZB-1701 column (30 m × 0.53 mm × 1 µm, Phenomenex, Torrance, CA, USA) containing a stationary phase composed of 14% phenyl cyanopropyl and 86% dimethylpolysiloxane. The temperature program began with an isothermal period at 50 °C for 5 min, followed by a ramp of 10 °C per minute up to 250 °C, where it was maintained for another 5 min. Additional analytical conditions included a detector temperature of 250 °C, an injector temperature of 230 °C, and a carrier gas flow rate of 0.8 mL/min. Based on the chromatographic results, the composition of all reaction mixtures (examining the effects of temperature, amount of catalyst, and reaction time) was determined. Subsequently, mass balances were calculated, allowing for the determination of geraniol conversion and the selectivity of its transformation into the desired products.

The conversion of geraniol was calculated using the following formula:

***Conversion of geraniol**** = (moles of geraniol that reacted/moles of geraniol initially introduced into the reactor)* × 100 [mol%]

The selectivity of geraniol transformation to oxidation products was calculated using the following formula:

***Product selectivity**** = (amount of a specific product/amount of geraniol reacted)* × 100 [mol%]

To identify the products formed during geraniol oxidation, selected post-reaction mixtures were additionally analyzed by gas chromatography–mass spectrometry (GC–MS). The analyses were performed using a Shimadzu Nexis GC-2030 gas chromatograph (Kyoto, Japan) coupled with a QP2020 NX mass spectrometer (Shimadzu Corporation, Kyoto, Japan) equipped with a SH-I-5MS capillary column (30 m × 0.25 mm × 0.25 μm, Shimadzu Corporation, Kyoto, Japan). Helium (99.999%) was used as the carrier gas at a constant flow rate of 0.94 mL min^−1^. The oven temperature was initially maintained at 40 °C for 2 min, increased to 300 °C at a rate of 10 °C min^−1^, and held at the final temperature for 2 min. The injector temperature was 250 °C. The MS transfer line and ion source temperatures were set at 250 °C and 200 °C, respectively. Mass spectra were acquired in the electron ionization (EI) mode at 70 eV over an *m*/*z* range of 35–500. The reaction products were identified by comparing their mass spectra with the NIST library database (Gaithersburg, MD, USA).

### 2.4. Kinetic Modeling

#### 2.4.1. Reaction Pathway

**Figure 2:**
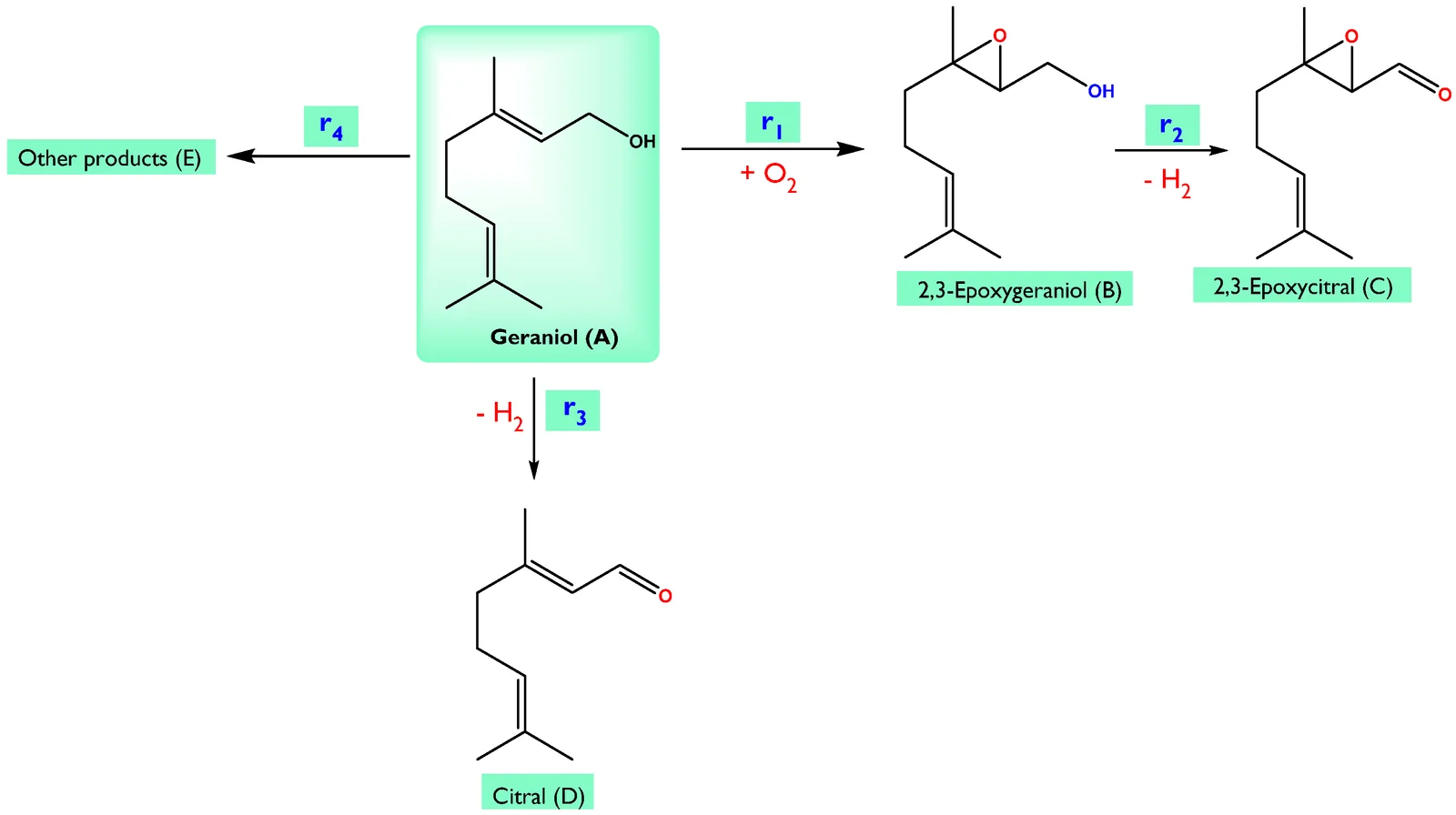
Reaction network for the geraniol transformation.

The proposed pathway for the oxidation of geraniol over the heterogeneous catalysts on P_POWDER and CP_KOH_850 is illustrated in Figure 2, and it is based on the experimental catalytic results. Geraniol (A) is mainly transformed through four primary routes: epoxidation with O_2_ to produce 2,3-epoxygeraniol (B), which can be further dehydrogenated to form 2,3-epoxycitral (C). In addition, citral (D) is generated via the oxidative dehydrogenation of geraniol. Very low formation was observed for products such as β-pinene and ocimenes from the dehydration of geraniol; linalool and nerol from geraniol isomerization; and thunbergol, a higher molecular weight compound formed through the dimerization of geraniol and/or its isomers. Finally, other products (E) are labeled to account for the conversion of geraniol into various side products.

#### 2.4.2. Kinetics

The reaction rate expressions are shown in Equations (1) and (2), which were derived assuming the surface reactions as irreversible, and k and K refer to the reaction rate constant and adsorption equilibrium constants, respectively. The detailed derivation of these kinetic equations was provided in the Supporting Information (Section S1). The reaction constants can be determined using the modified Arrhenius equation, Equation (3), as described in the literature [19,20]. Here, k_ref_ represents the reaction rate constant at a reference temperature (T_ref_), E_a_ is the activation energy, and R is the gas constant. On the other hand, the adsorption constants can be assumed constant within the temperature range [21].

(1) $$r_{i}=\frac{k_{i}C_{A}}{1+K_{A}C_{A}+K_{B}C_{B}+{K_{C}C}_{C}+K_{D}C_{D}+{K_{E}C}_{E}}\;\;\left(i=1,3,4\right)$$

(2) $$r_{2}=\frac{k_{2}C_{B}}{1+K_{A}C_{A}+K_{B}C_{B}+{K_{C}C}_{C}+K_{D}C_{D}+{K_{E}C}_{E}}$$

(3) $$k=k_{ref}e^{-\frac{E_{a}}{R}\left(\frac{1}{T}-\frac{1}{T_{ref}}\right)}$$

The mole balance for each species in the batch reactor is given by Equation (4), where C_j_ is the molar concentration of species j, m_cat_ is the catalyst mass, V is the reaction volume, and R_j_ is the generation rate for species j, as shown in Table 1.

(4) $$\frac{dC_{j}}{dt}=\frac{m_{cat}}{V}R_{j}$$

The estimation of parameters was performed through nonlinear regression using the ModEst modeling and parameter estimation software (v1, 1994) [22], as reported elsewhere [19], where the objective function (Equation (5)) is minimized through the Levenberg–Marquardt algorithm.

(5) $$O.F=\sum_{j}^{N_{com}}\sum_{i=1}^{N_{obs}}{\left(C_{j,i,Exp}-C_{j,i,Calc}\right)}^{2}$$

#### 2.4.3. Determination of the Acid Sites Concentration in Samples of P_POWDER, CP_KOH_750, CP_KOH_800, CP_KOH_850 Catalysts

The acid sites concentration in P_POWDER, CP_KOH_750, CP_KOH_800, CP_KOH_850 was determined using the titration method described by Vilcocq et al. [23]. In this method, 40 mg of catalytic porous material was added to 20 cm^3^ of 0.01 M solution of NaOH. The mixture was next shaken at room temperature for 2 h. The material was then filtered, and the pH of the filtrate was determined by titration with 0.01 M solution of HCl in the presence of phenolphthalein as the indicator. The acid site concentration, Ns, was established according to Equation (6):

Ns = ([OH−]o − [OH−]2h) × V/m(6)

where

[OH−] = the hydroxide group molar concentration determined by the titration (mol/dm^3^),

V = the volume of NaOH solution added to porous material sample, and

m = the mass of porous material sample

## 3. Results and Discussion

### 3.1. Characteristics of Materials for Catalytic Studies in the Form of Dried Powder from Pelargonium zonale and Activated Carbons from Pelargonium zonale Obtained at the Carbonization Temperatures: 750, 800, and 850 °C

Samples named dried powder from *Pelargonium zonale* (P_POWDER), activated carbon from *Pelargonium zonale* at 750 °C (CP_KOH_750), activated carbon from *Pelargonium zonale* at 800 °C (CP_KOH_800), and activated carbon from *Pelargonium zonale* at 850 °C (CP_KOH_850) were subjected to instrumental analyses.

**Figure 3:**
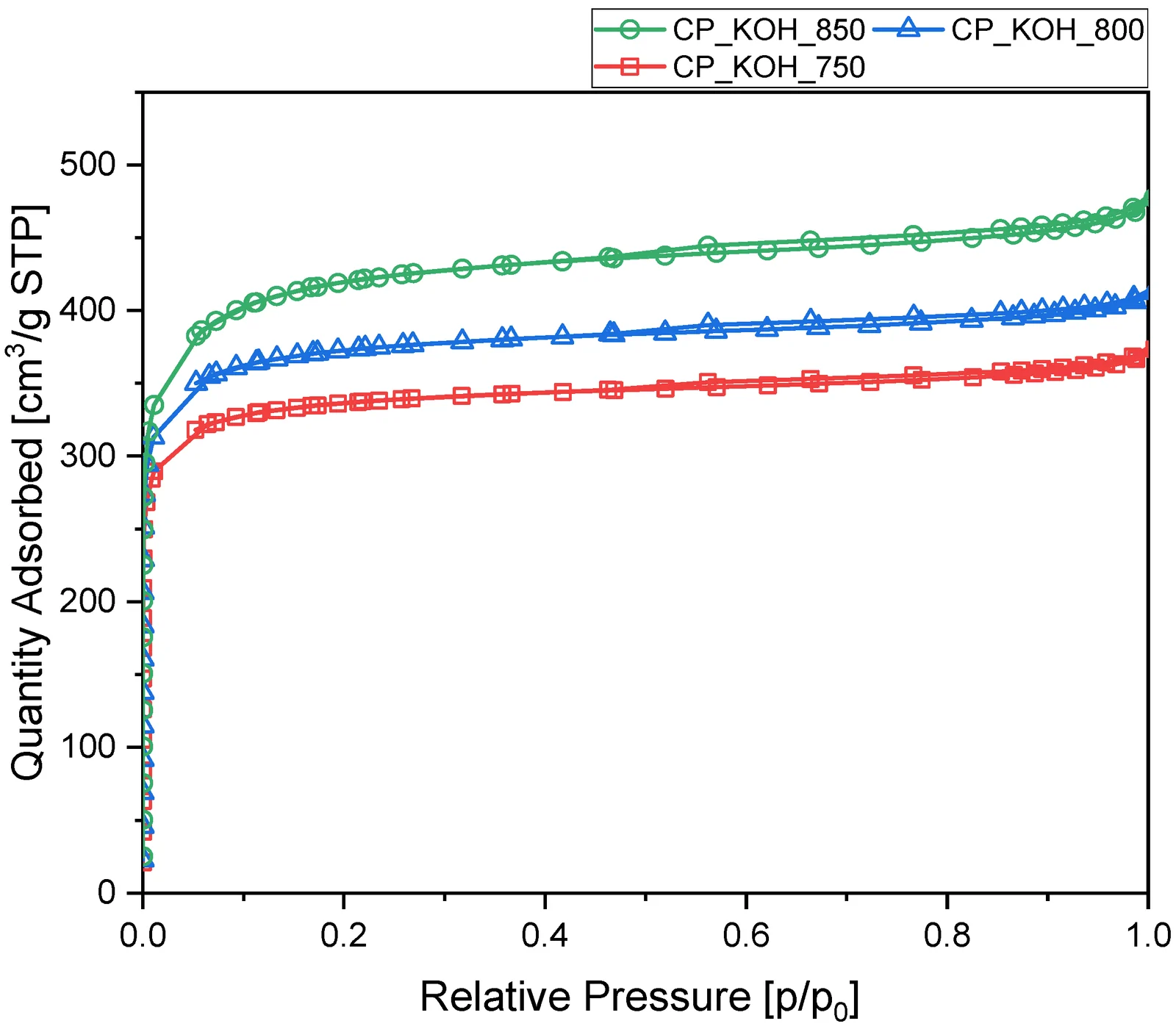
Nitrogen adsorption–desorption isotherms of CP_KOH_750, CP_KOH_800, CP_KOH_850.

Figure 3 presents the nitrogen adsorption–desorption isotherms for all the samples, which exhibit isotherms of Type I(b) according to the IUPAC classification, characteristic of predominantly microporous materials, especially activated carbons, with the possible presence of narrow small mesopores.

At low relative pressures (p/p_0_ < 0.05), all samples show a steep uptake of nitrogen, confirming the formation of a well-developed microporous network. The initial adsorption increases systematically with activation temperature, indicating progressive pore development under stronger KOH etching conditions. The sample activated at 850 °C (CP_KOH_850) reaches the highest adsorption capacity in the entire pressure range, resulting in the largest overall pore volume (Table 2) and the most extensive micropore development.

In the intermediate pressure region (0.05 < p/p_0_ < 0.3), the isotherms gradually level off, reflecting the filling of wider micropores and the onset of adsorption in transitional ultramicropores. Only a slight hysteresis loop is observed, indicating that the materials remain predominantly microporous, although the presence of narrow mesopores is also indicated.

At higher relative pressures (p/p_0_ > 0.3), all isotherms display a slight but noticeable upward trend, particularly for the CP_KOH_850 material. This behavior is attributed to the presence of a smaller extent of small mesopores, which become more pronounced with increasing activation temperature. Their development is consistent with the progressive widening of micropores due to intensified chemical activation. Due to the negligible content of mesopores and macropores, no significant increase in nitrogen adsorption is observed as p/p_0_ approaches 1, which would otherwise be associated with capillary condensation in larger pores.

Overall, the results clearly demonstrate that higher activation temperatures promote a larger specific surface area and enlarged pore volume, as summarized in Table 2, with CP_KOH_850 exhibiting 1578 m^2^ g^−1^ and 0.751 cm^3^ g^−1^. These trends correlate with the stronger activation effect of KOH at elevated temperatures, leading to enhanced carbon burn-off and formation of a more open porous structure.

**Figure 4:**
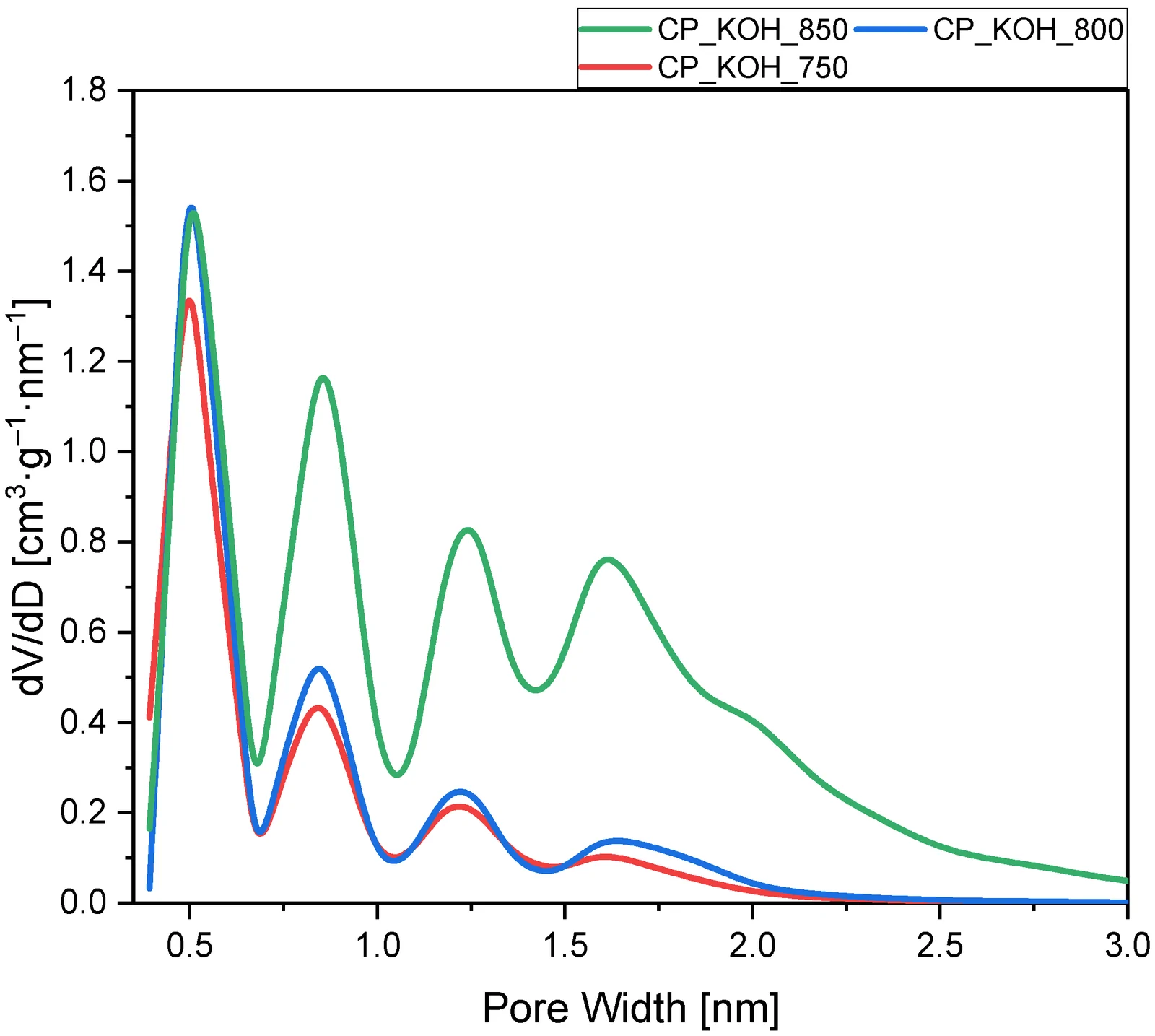
Pore size distribution of CP_KOH_750, CP_KOH_800, CP_KOH_850, calculated via the DFT method using N_2_ sorption at a temperature of 77 K.

Figure 4 shows the pore size distributions obtained by DFT method for all the activated carbons. All samples exhibit a strong contribution from ultramicropores and narrow micropores, although the relative intensity and position of the maxima change noticeably with increasing activation temperature. (We adopted the IUPAC classification, according to which micropores are pores with widths smaller than 2 nm, whereas ultramicropores are a subset of micropores with widths smaller than 0.7 nm.)

For the material activated at 750 °C, the dominant peak appears at approximately 0.5 nm, indicating a high fraction of very narrow pores that are typically responsible for adsorption at low relative pressures. The distribution remains relatively simple, consistent with a less extensively developed pore structure. In the sample obtained at 800 °C, the intensity of the primary ultramicropore peak decreases, while additional maxima emerge in the 0.7–1.0 nm transition region. This shift explains partial widening of the narrowest pores due to stronger chemical activation, in line with the increase in adsorption capacity previously observed.

The material activated at 850 °C displays the most complex and heterogeneous pore structure. Multiple peaks of significantly higher intensity are observed across the entire micropore range, particularly between 0.7 and 1.5 nm. The presence of several well-defined maxima reflects an advanced degree of pore widening and interconnected pore development, characteristic of aggressive KOH activation at elevated temperatures.

The pore size distributions therefore confirm that higher activation temperatures promote not only an increase in total pore volume but also a larger heterogeneity within the micropore network, especially in the pore sizes that contribute to enhanced adsorption at higher relative pressures.

Table 2 summarizes the key textural parameters of the activated carbons. Each sample was synthesized and characterized once; therefore, the values reported in Table 2 correspond to single measurements, and no experimental uncertainties or standard deviations are reported.

A clear and systematic increase in the specific surface area (SSA) and total pore volume is observed as the activation temperature rises from 750 to 850 °C. These trends reflect the progressive intensification of the carbon–KOH reaction, which enhances pore development and increases the overall degree of burn-off.

The SSA increases from 1312 m^2^ g^−1^ for CP_KOH_750 to 1578 m^2^ g^−1^ for CP_KOH_850, consistent with the widening and increasing of microporosity revealed by the DFT distributions. Similarly, the total pore volume rises from 0.587 to 0.751 cm^3^ g^−1^, indicating that higher temperatures promote not only the generation of new pores but also the expansion of existing ones.

The micropore volume also follows this pattern, reaching 0.533 cm^3^ g^−1^ for the 850 °C material. However, the microporosity decreases slightly at the highest activation temperature (71%), compared with the 750–800 °C samples (75–76%). This effect is consistent with the generation of additional wider micropores and narrow mesopores, which contribute to V_tot_ but reduce the relative share of strictly microporous structures.

Overall, the textural data confirm that higher activation temperatures lead to a more accessible and structurally diverse pore network, in agreement with both adsorption isotherms and DFT-derived pore size distributions. The combined results demonstrate that activation at 850 °C produces the most developed hierarchical microporous system among the investigated materials.

**Figure 5:**
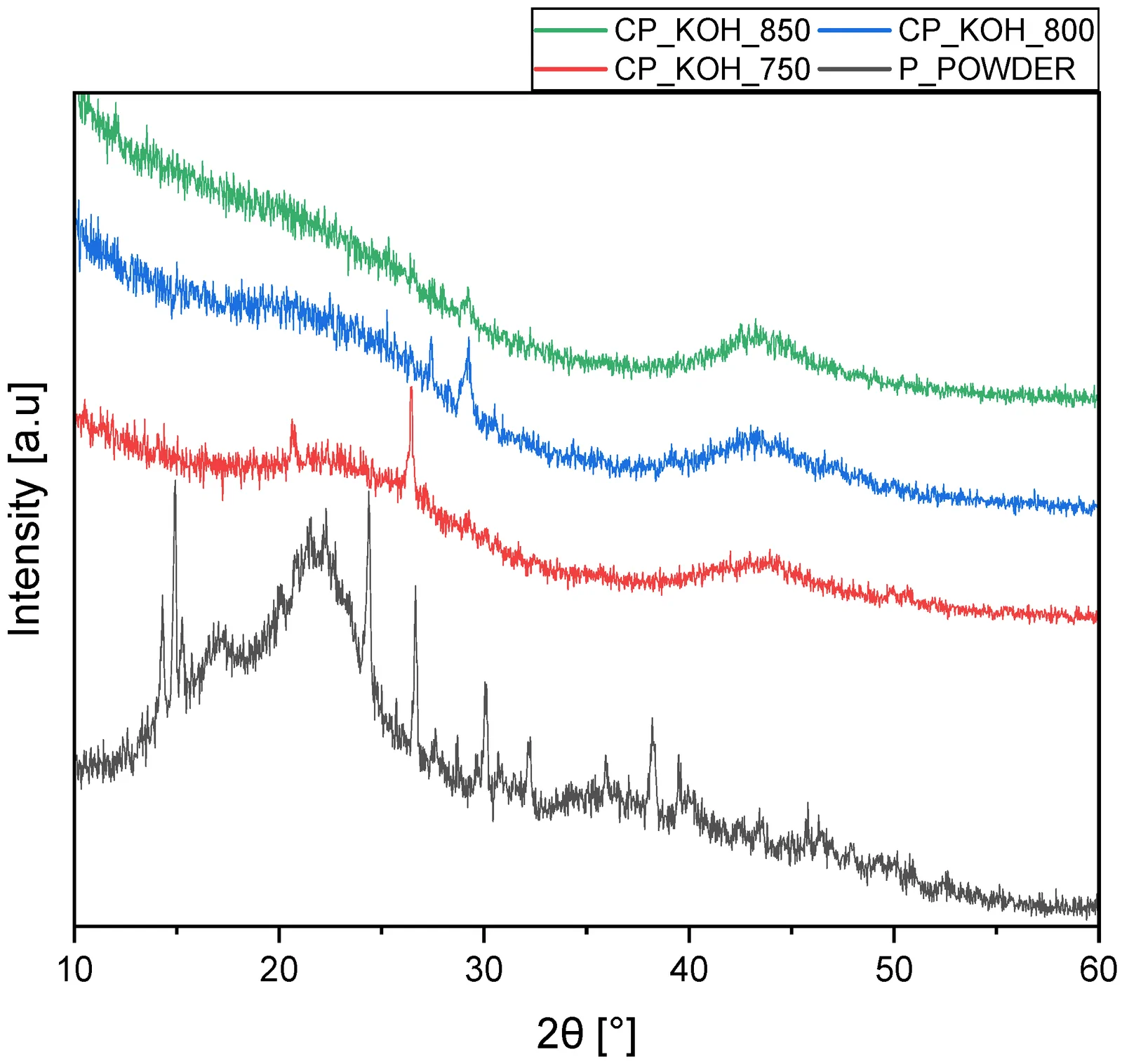
XRD patterns of P_POWDER, CP_KOH_750, CP_KOH_800, CP_KOH_850.

Figure 5 presents the XRD patterns of the raw pelargonium powder (P_POWDER) and the carbons activated with KOH at 750, 800, and 850 °C.

The XRD pattern of the pelargonium precursor (P_POWDER) contains several sharp diffraction peaks corresponding to crystalline inorganic phases naturally present in plant tissues. Peaks characteristic of calcium oxalate hydrate (JCPDS 16-0379) and silica (JCPDS 85-0335) were identified. The presence of these phases confirms that the raw biomass contains a significant amount of mineral components, which are removed or transformed during carbonization and KOH activation.

A clear transformation of the diffraction profiles is observed when moving from the biomass to the carbonized and activated materials. The sharp reflections originating from crystalline inorganic phases in the precursor disappear, and the activated samples exhibit diffraction patterns typical of disordered carbon structures. All KOH-activated carbons show broad and diffuse diffraction bands located at: (i) 22–25° 2θ, which corresponds to the (002) reflection of turbostratic carbon domains (nearly invisible), and (ii) 42–45° 2θ, associated with the (100)/(101) reflections of poorly ordered graphene layers [24,25].

The presence of these broad peaks confirms the amorphous nature of the obtained carbons and the absence of long-range ordering characteristic of graphitic materials. The significant peak broadening indicates small graphene-like domains and a high density of defects, which is a typical consequence of aggressive chemical activation [26].

The disappearance of the sharp peaks observed in the precursor confirms the effective removal or transformation of mineral components during carbonization and activation. The obtained diffraction patterns are characteristic of KOH-activated carbons with well-developed microporosity and turbostratic structural features [27,28], consistent with the BET and DFT analyses.

Table 3 summarizes the elemental composition of the pelargonium precursor (P_POWDER) and the carbons activated with KOH at 750, 800, and 850 °C. The precursor contains relatively high amounts of calcium, which is consistent with the presence of naturally occurring mineral components in plant tissues, as previously confirmed by XRD analysis.

A pronounced decrease in the concentrations of Ca, K, Cl, and P is observed after activation. This reduction reflects the removal or transformation of inorganic phases during carbonization and subsequent KOH activation, which is known to effectively leach out mineral components or convert them into soluble forms. Calcium content decreases from 6.30 wt% in the precursor to 2.60–3.73 wt% in the activated samples, while potassium is almost completely removed, dropping from 4.44 wt% to below 0.37 wt%. A similar trend is observed for chlorine and phosphorus, whose concentrations fall to trace levels in all activated carbons.

Silicon behaves differently: its relative content increases in the activated carbons compared with the precursor. This effect is consistent with the concentration of thermally stable siliceous species (e.g., amorphous SiO_2_) due to carbon burn-off during activation. The highest Si content observed in CP_KOH_750 and CP_KOH_850 suggests that silica is more resistant to the activation conditions than the other inorganic components.

Overall, the XRF results confirm the substantial removal of mineral matter during KOH activation and highlight the persistence of silicon-containing phases. These findings align well with the XRD data, which showed the disappearance of crystalline mineral reflections and the dominance of amorphous carbon structures in the activated materials.

**Figure 6:**
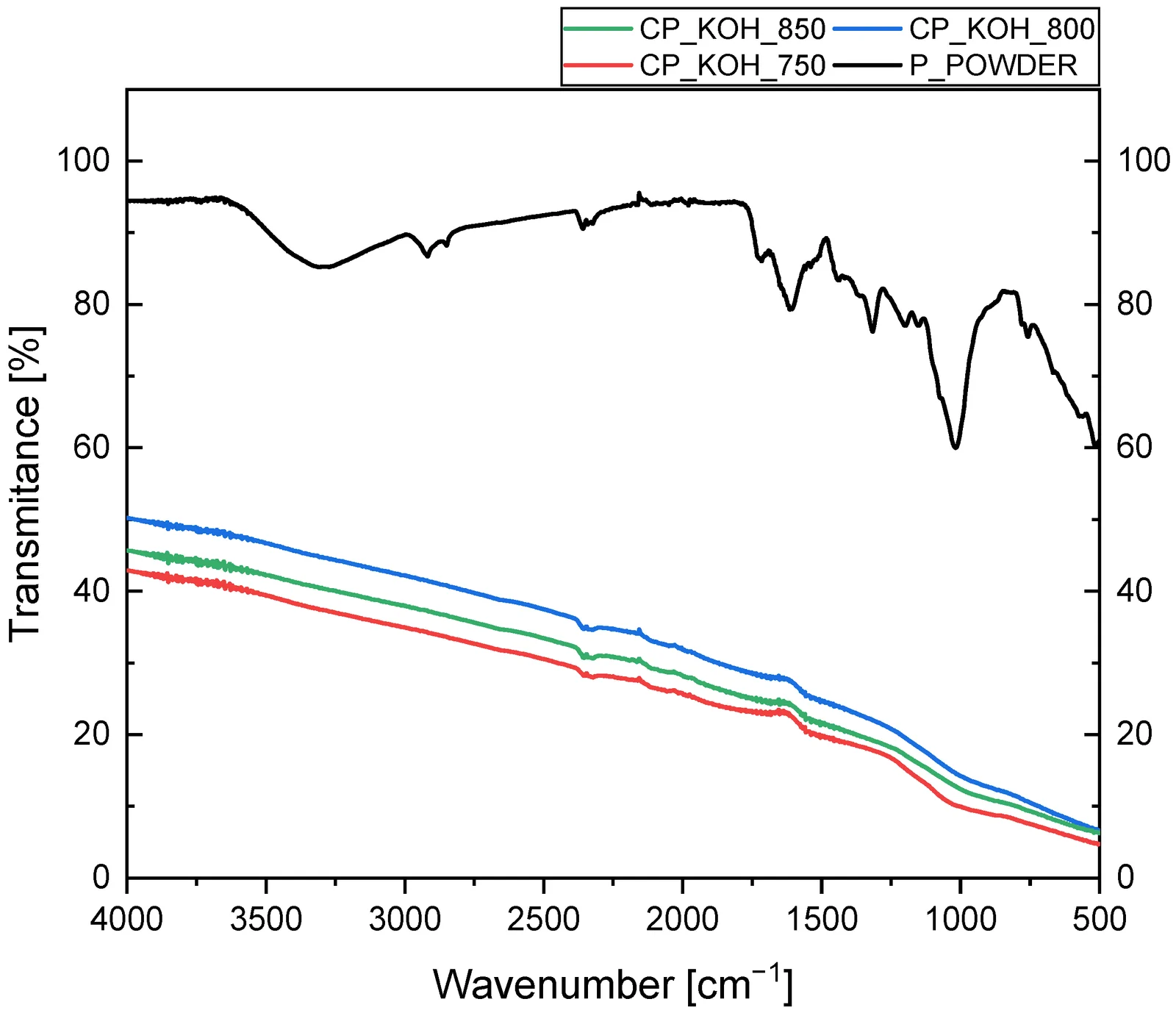
FTIR spectra of P_POWDER, CP_KOH_750, CP_KOH_800, CP_KOH_850.

The FTIR spectra of P_POWDER, CP_KOH_750, CP_KOH_800 and CP_KOH_850 are presented in Figure 6.

The FTIR spectra of all the materials are presented in Figure 6. The spectrum of the raw pelargonium powder differs significantly from those of the activated carbons, indicating extensive chemical transformations occurring during the processes of carbonization and KOH activation.

For P_POWDER, a broad absorption band centered at approximately 3307 cm^−1^ is assigned to O–H stretching vibrations associated with hydroxyl groups and adsorbed water. The band observed at approximately 2918 cm^−1^ corresponds to CH_2_ and CH_3_ stretching vibrations of aliphatic structures. A distinct absorption band at around 1716 cm^−1^ can be attributed to C=O stretching vibrations originating from carbonyl-containing structures. The signal near 1615 cm^−1^ is associated with aromatic C=C stretching vibrations originating from lignin-derived aromatic structures and condensed aromatic carbon domains. Additional bands observed in the region 1200–1000 cm^−1^ are characteristic of C–O stretching vibrations present in polysaccharides and other oxygen-containing organic structures. The intense band observed at approximately 1019 cm^−1^ is mainly attributed to C–O stretching vibrations, although a contribution from Si–O vibrations cannot be excluded due to the presence of silicon-containing phases detected by XRF and XRD [29].

After carbonization and chemical activation with KOH, substantial changes in the FTIR spectra are observed. The characteristic bands associated with lignocellulosic components decrease markedly or disappear almost completely, indicating the decomposition of the original plant constituents and the formation of a more condensed carbon-rich structure. The spectra of CP_KOH_750, CP_KOH_800, and CP_KOH_850 still exhibit weak residual absorptions in the regions corresponding to O–H, C=O/C=C, and C–O vibrations, although their intensities are markedly lower than those observed for P_POWDER [30,31]. The relatively featureless character of the activated carbon spectra is typical of highly carbonized materials and results from the progressive development of condensed aromatic structures, which produce a broad spectral background and reduce the intensity of individual vibrational bands. The significant reduction in the intensity of O–H, C–H, C=O, and C–O bands confirms progressive dehydration, dehydrogenation, and aromatization of the biomass during thermal treatment, leading to the formation of increasingly condensed carbonaceous structures [29,30]. The similarity of the spectra obtained for CP_KOH_750, CP_KOH_800, and CP_KOH_850 suggests that increasing activation temperature does not substantially alter the nature of the surface functional groups but primarily affects the textural properties of the materials, as confirmed by BET and DFT analyses.

**Figure 7:**
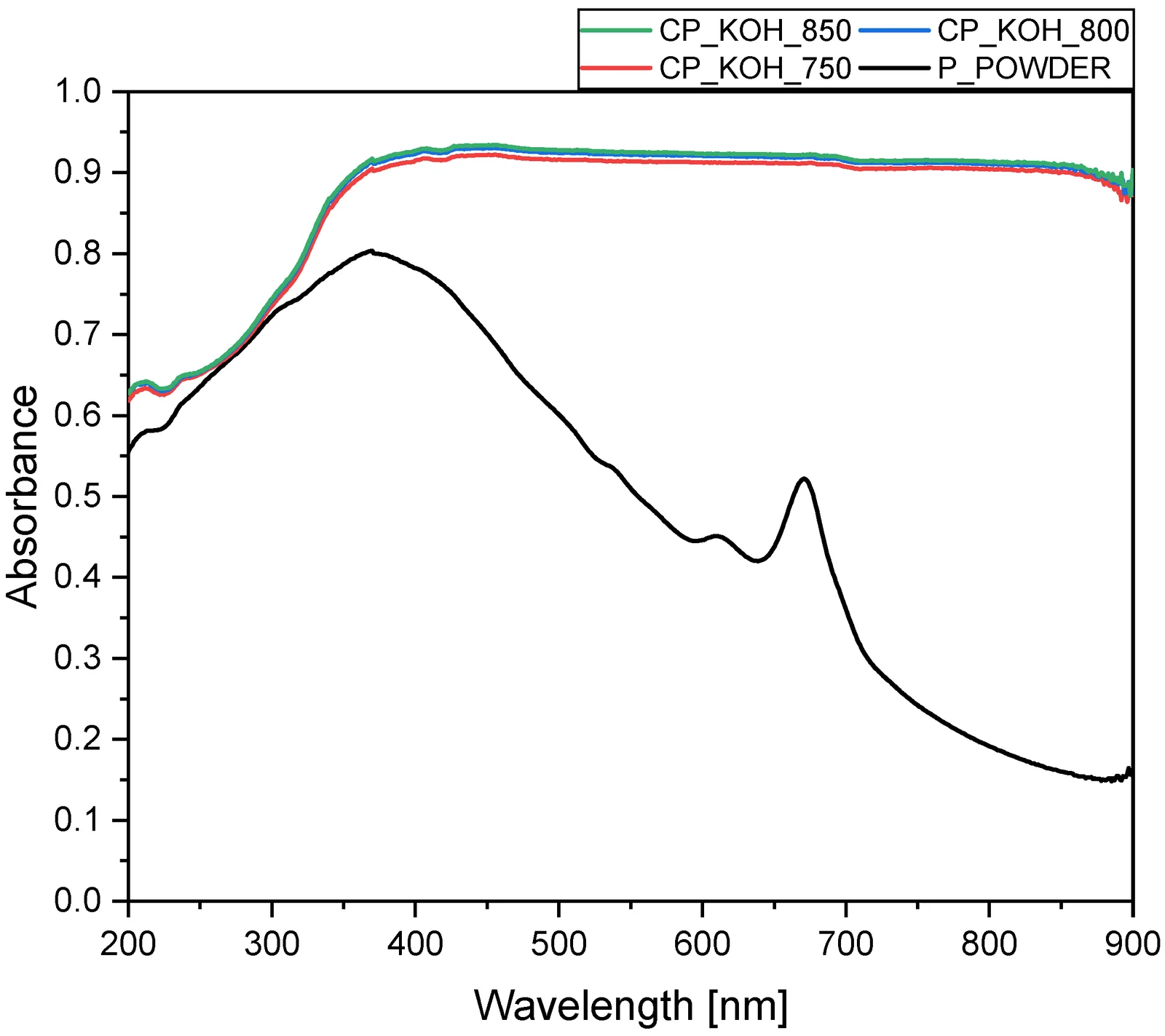
UV–Vis DRS of P_POWDER, CP_KOH_750, CP_KOH_800, CP_KOH_850.

The UV–Vis diffuse reflectance spectra (DRS) of the samples are presented in Figure 7. The spectrum of the raw pelargonium powder differs markedly from those of the activated carbons, reflecting the substantial chemical transformations occurring during carbonization and KOH activation.

The P_POWDER sample exhibits a broad absorption band extending from approximately 250 to 500 nm, with a maximum near 380–400 nm. This absorption region is characteristic of lignocellulosic biomass and is mainly associated with aromatic structures originating from lignin as well as various oxygen-containing organic compounds present in plant tissues [32]. In addition, a distinct absorption feature observed at approximately 670 nm can be attributed to chlorophyll-related pigments naturally occurring in dried *Pelargonium zonale* biomass [33].

After carbonization and chemical activation, the spectra of CP_KOH_750, CP_KOH_800, and CP_KOH_850 differ significantly from that of the precursor material. The characteristic absorption bands associated with native plant constituents disappear, indicating extensive decomposition of lignocellulosic structures and the formation of carbon-rich materials [34]. The activated carbon samples exhibit nearly featureless spectra with high and relatively constant absorbance throughout the investigated wavelength range. Such behavior is typical of highly carbonized materials containing extended aromatic and conjugated carbon domains, where broad electronic transitions overlap and mask individual absorption features [34,35].

Only minor differences are observed between CP_KOH_750, CP_KOH_800, and CP_KOH_850, suggesting that increasing activation temperature does not substantially alter the nature of the electronic structure of the carbon matrix. Instead, the primary effect of temperature is related to the development of porosity and textural properties, as confirmed by BET and DFT analyses.

**Figure 8:**
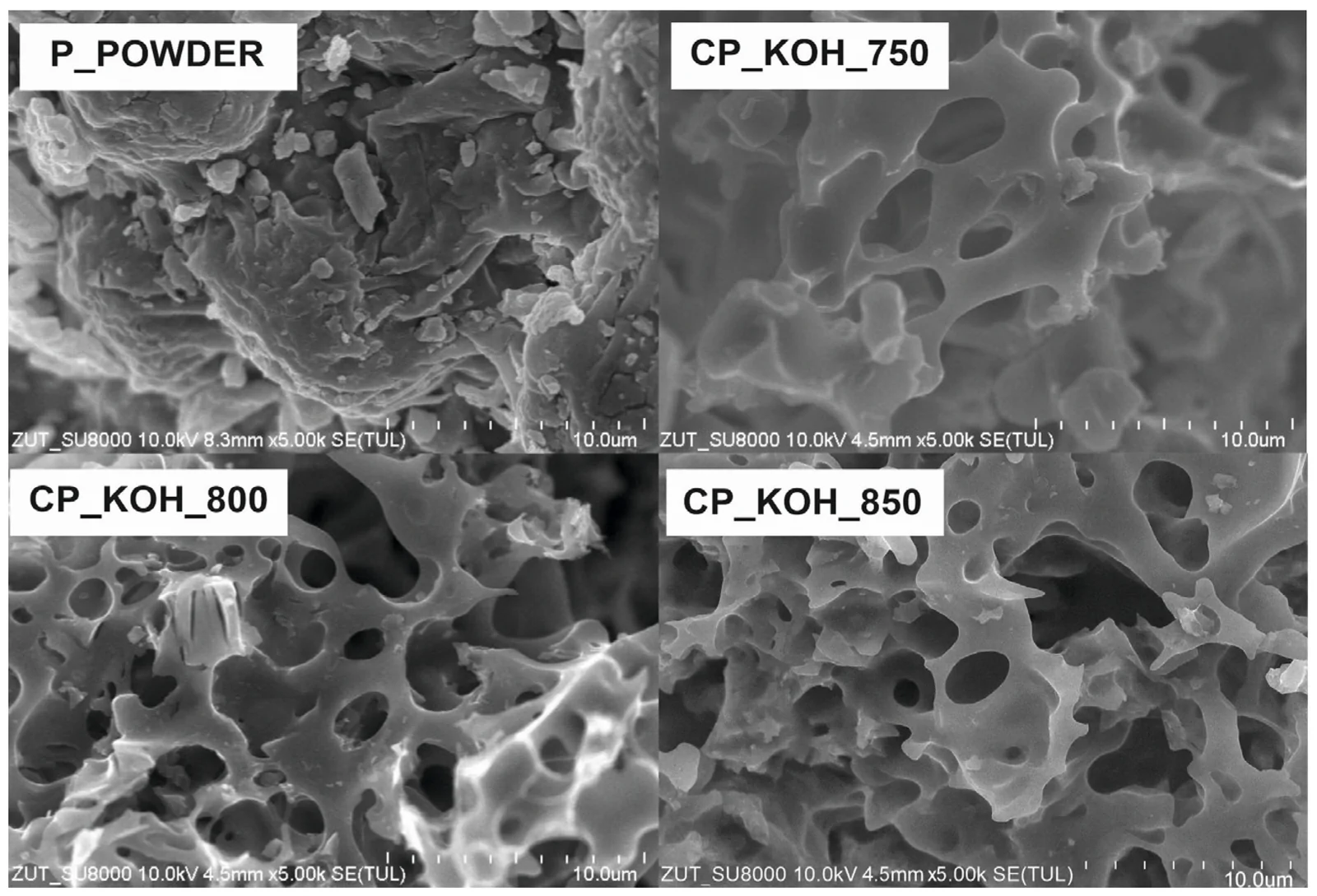
The SEM images of P_POWDER, CP_KOH_750, CP_KOH_800, CP_KOH_850 at magnifications 5k.

Figure 8 shows SEM images of pelargonium powder before activation (P_POWDER) and the materials subjected to KOH activation at 750, 800, and 850 °C.

The structure of the precursor exhibits a compact, irregular arrangement of aggregates with smooth surfaces and without any clearly developed porosity. Only small cracks and fissures are visible, originating from the natural structure of the plant biomass; however, they do not form an ordered pore network characteristic of carbon materials after activation. In the case of the KOH-activated materials, a pronounced change in morphology is observed. The resulting activated carbons exhibit a clearly open, sponge-like structure with numerous cavities and openings of various shapes, arising from the development of the pore system through the reaction between KOH and carbon. The structure is considerably more fragmented compared with the precursor. In all materials, numerous macropores are visible, which are the only pore category that can be visualized by SEM. Therefore, the samples activated at 750, 800, and 850 °C display a similar overall morphological appearance, even though their textural properties differ significantly, as confirmed by adsorption measurements.

Since surface acidity may influence the catalytic performance of carbon-based materials, the concentration of acid sites was determined for P_POWDER and the activated carbons obtained from *Pelargonium zonale*. The results are presented in Table 4.

As shown in Table 4, the highest concentration of acid sites was observed for P_POWDER (2.37 mmol g^−1^), whereas all activated carbon samples exhibited significantly lower values, ranging from 0.80 to 0.96 mmol g^−1^. The substantial decrease in acidity after carbonization and KOH activation can be attributed primarily to the decomposition of oxygen-containing surface functional groups naturally present in the plant biomass, including hydroxyl, carboxyl, and other acidic moieties. This interpretation is consistent with the FTIR results, which revealed a pronounced reduction in the intensity of bands associated with oxygen-containing functionalities after thermal treatment. In addition, XRF analysis demonstrated substantial changes in the mineral composition of the materials during activation, which may also influence their surface chemical properties.

Among the activated carbons, CP_KOH_750 exhibited the highest acid site concentration (0.96 mmol g^−1^), whereas the lowest value was determined for CP_KOH_800 (0.80 mmol g^−1^). The relatively similar acidity values obtained for the activated carbons suggest that increasing activation temperature affects the textural properties of the materials to a greater extent than their overall surface acidity. It should be noted that the titration method applied in this study provides only the total concentration of acid sites and does not distinguish between Brønsted and Lewis acid sites. Therefore, the present results do not allow assessment of the individual contribution of different acid site types to the catalytic behaviour of the investigated materials. Since acid sites may participate in oxidation, isomerization, and dehydration reactions, the observed differences in acidity may contribute to variations in catalytic activity and product distribution during geraniol oxidation.

Overall, the physicochemical characterization confirms that KOH activation profoundly modifies the structure of *Pelargonium zonale*-derived materials. Compared with the precursor biomass (P_POWDER), the activated carbons exhibit a highly developed porous structure, significantly increased specific surface area, reduced mineral content, lower surface acidity, and a predominantly amorphous carbon framework. FTIR and UV–Vis analyses additionally indicate extensive decomposition of the original lignocellulosic constituents and the formation of condensed aromatic carbon structures. The acid site measurements further demonstrate that carbonization and KOH activation lead to a substantial reduction in the concentration of acidic surface functionalities. Among the investigated materials, CP_KOH_850 displays the most developed textural properties, characterized by the highest specific surface area, pore volume, and the wider microporosity distribution. These structural and chemical features are expected to influence the catalytic performance of the materials in the oxidation of geraniol. Therefore, the catalytic experiments were undertaken to determine whether the observed differences in textural properties, surface chemistry, and acidity translate into differences in catalytic activity and product selectivity during geraniol oxidation.

### 3.2. Catalytic Research on the Catalyst from Dried Powder of Pelargonium zonale and Activated Carbons from Pelargonium zonale

The catalytic performance of the four *Pelargonium zonale*-derived materials was evaluated in the oxidation of geraniol with molecular oxygen. The influence of three reaction parameters was investigated: reaction temperature, catalyst amount, and reaction time. Geraniol conversion and product distribution were used to assess catalytic behaviour.

**Figure 9:**
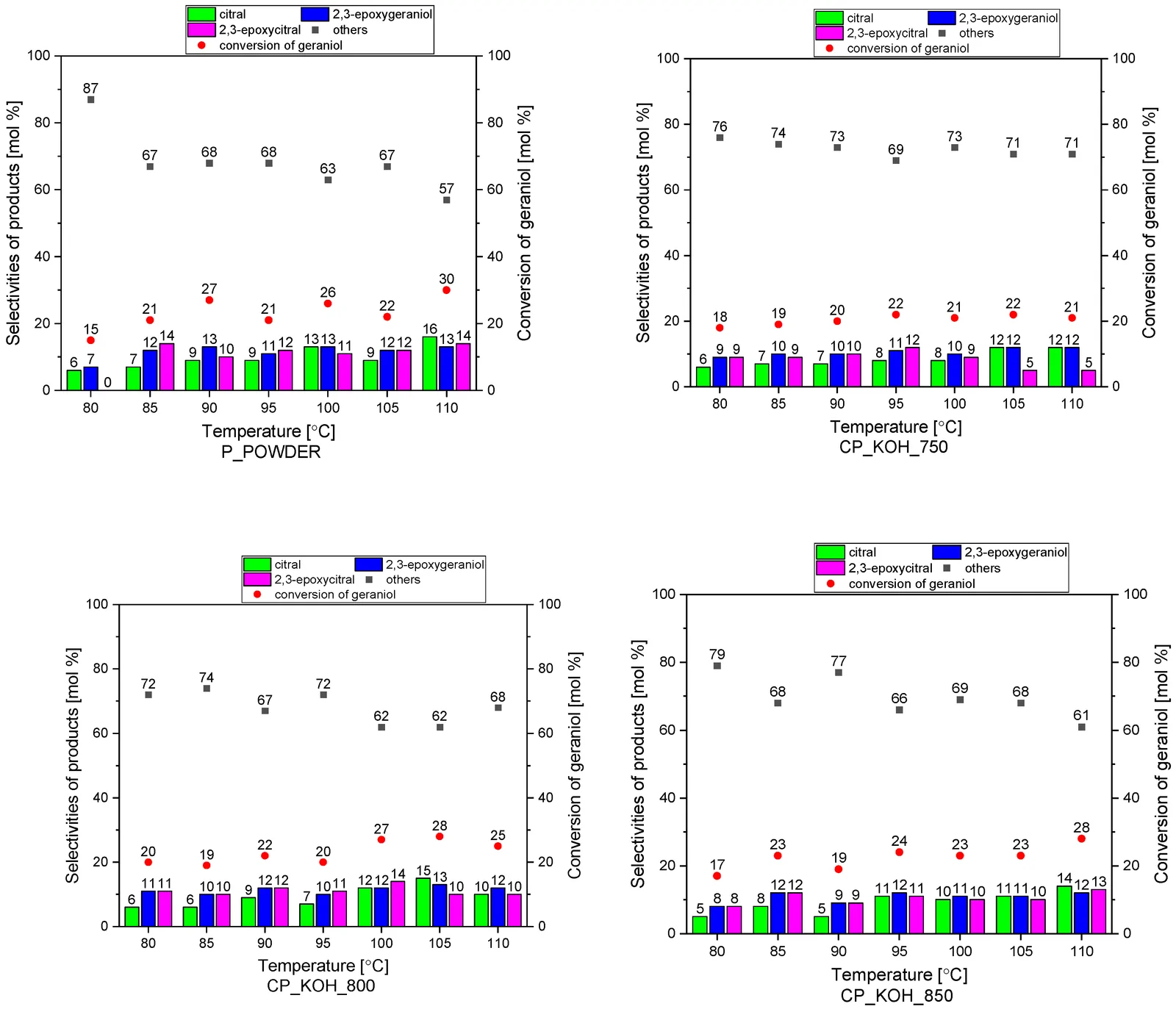
Effect of reaction temperature (80–110 °C) on geraniol conversion and product selectivity during geraniol oxidation over P_POWDER, CP_KOH_750, CP_KOH_800, and CP_KOH_850. Catalyst amount: 1 wt%; reaction time: 5 h.

**Figure 10:**
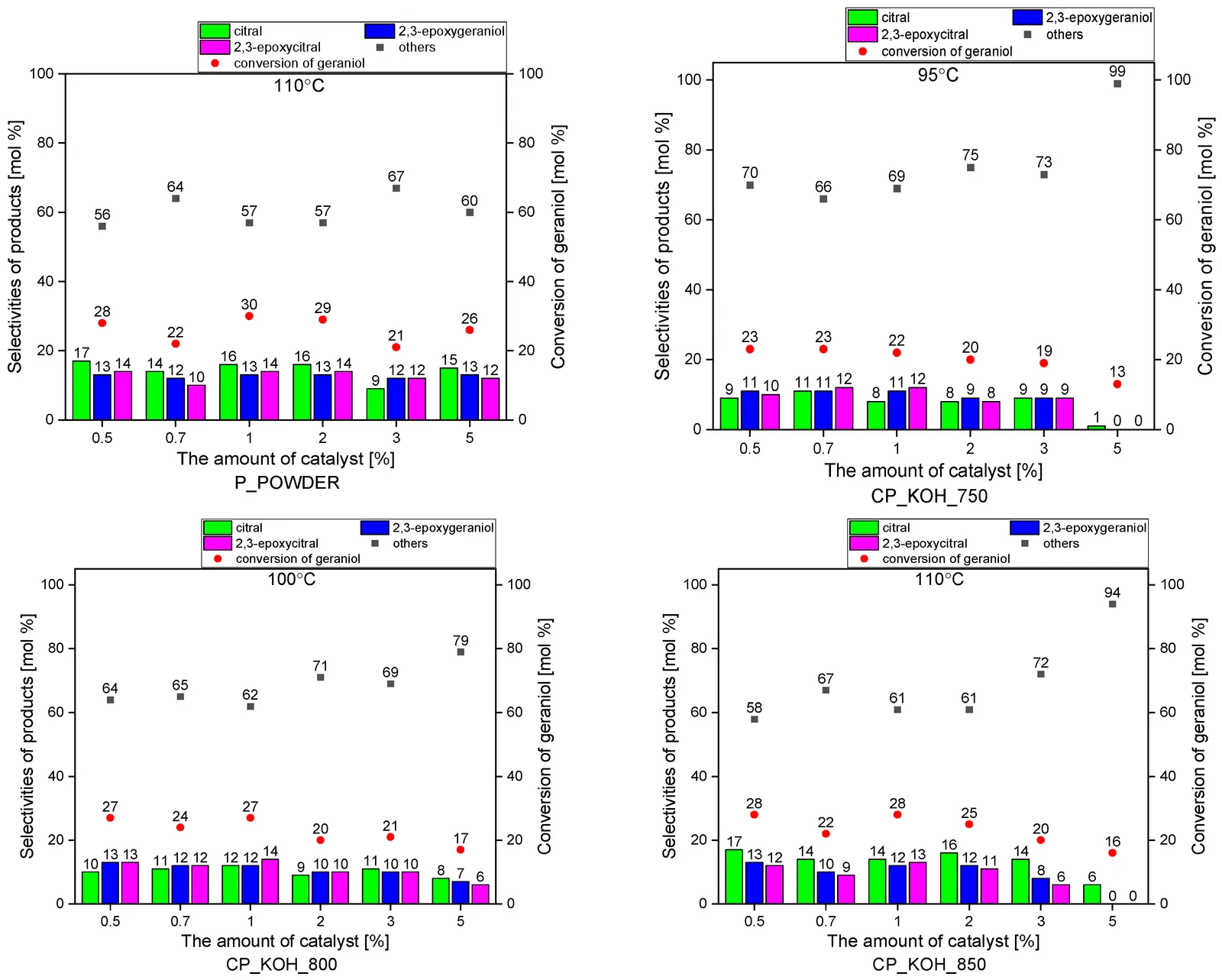
Effect of the amount of catalyst (0.5–5 wt%) on geraniol conversion and product selectivity during geraniol oxidation over P_POWDER, CP_KOH_750, CP_KOH_800, and CP_KOH_850. Reaction temperatures: 110 °C for P_POWDER, 95 °C for CP_KOH_750, 100 °C for CP_KOH_800, and 110 °C for CP_KOH_850. Reaction time: 5 h.

**Figure 11:**
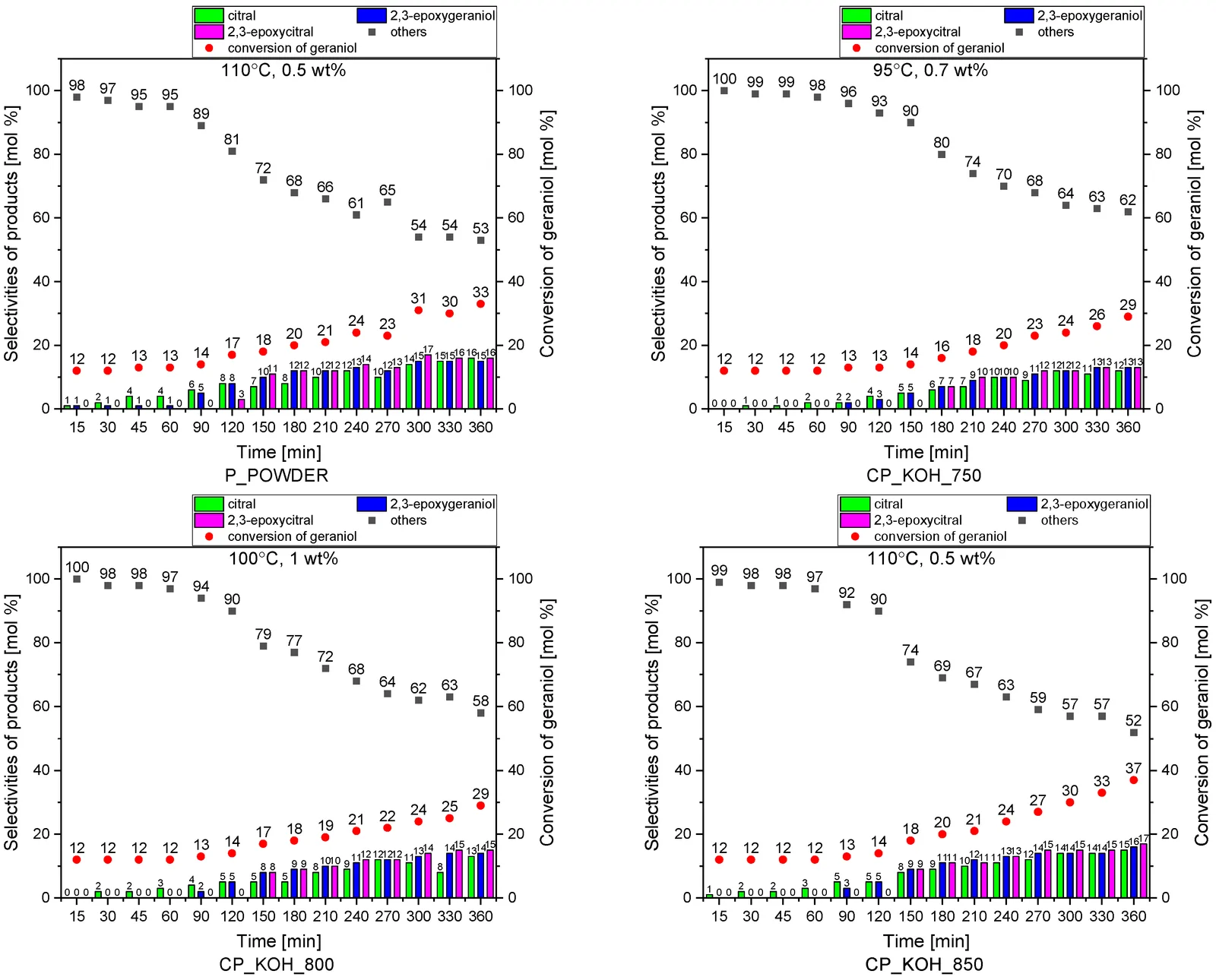
Effect of reaction time (15–360 min) on geraniol conversion and product selectivity during geraniol oxidation over P_POWDER, CP_KOH_750, CP_KOH_800, and CP_KOH_850. The reaction was performed under the optimal temperature and catalyst amount determined for each catalyst (110 °C and 0.5 wt%, 95 °C and 0.7 wt%, 100 °C and 1 wt%, and 110 °C and 0.5 wt%, respectively).

The main identified oxidation products were citral, 2,3-epoxygeraniol, and 2,3-epoxycitral. In addition, several minor products were detected, including β-pinene, ocimenes, nerol, linalool, 6,11-dimethyl-2,6,10-dodecatrien-1-ol, thunbergol, and 1,6-octadien-3-ol-3,7-dimethyl formate. Since each of these compounds was formed in relatively low individual amounts, they were grouped together as “others” in Figure 9, Figure 10 and Figure 11. It should be emphasized that although the individual selectivity of each minor product was low, their cumulative selectivity was high, indicating that the oxidation of geraniol over the studied materials proceeds through several parallel and consecutive reaction pathways. The effect of temperature was first investigated in the range of 80–110 °C using 1 wt% catalyst and a reaction time of 5 h. The results are presented in Figure 9.

Comparison of the results presented in Figure 9 shows that reaction temperature influenced geraniol conversion; however, no strictly monotonic dependence was observed over the investigated temperature range. For P_POWDER, geraniol conversion generally increased from 15 mol% at 80 °C to 30 mol% at 110 °C, although some fluctuations were observed at intermediate temperatures. For the activated carbons, the conversion varied within relatively narrow ranges (18–22 mol% for CP_KOH_750, 19–28 mol% for CP_KOH_800, and 17–28 mol% for CP_KOH_850). Considering that each catalytic experiment was performed independently using a fresh catalyst sample and that each reaction mixture was analyzed three times by GC, small differences between neighbouring temperature points should be interpreted with caution, as they may reflect the inherent variability of catalytic experiments and analytical measurements rather than a true kinetic trend.

A more pronounced influence of temperature was observed on product selectivity. For all catalysts, increasing temperature generally promoted the formation of citral. The selectivity to citral increased from 6 to 16 mol% for P_POWDER, from 6 to 12 mol% for CP_KOH_750, and from 5 to 14 mol% for CP_KOH_850. For CP_KOH_800, the selectivity to citral reached a maximum of 15 mol% at 105 °C, followed by a decrease to 10 mol% at 110 °C. A similar tendency was observed for 2,3-epoxygeraniol, although the increase in selectivity was less pronounced. The behaviour of 2,3-epoxycitral was more dependent on the catalyst type. For P_POWDER and CP_KOH_850, the selectivity to this product generally increased with increasing temperature, reaching 14 and 13 mol%, respectively. In contrast, CP_KOH_750 and CP_KOH_800 exhibited maximum selectivities of 12 mol% at 95 °C and 14 mol% at 100 °C, respectively, followed by a decrease at higher temperatures.

The selectivity toward the remaining products, grouped as “others”, was considerably higher than that of the main oxidation products and ranged from 57 to 87 mol%, depending on the catalyst and reaction temperature. Although the individual selectivities of the compounds included in this fraction were low, their cumulative contribution indicates that geraniol oxidation proceeded through several parallel reaction pathways under the applied reaction conditions. In general, increasing temperature reduced the selectivity toward this fraction and promoted the formation of the identified oxidation products, particularly citral. Nevertheless, the occurrence of secondary reactions, including condensation and oligomerization processes leading to the formation of higher-molecular-weight products, cannot be excluded, particularly at elevated reaction temperatures.

The catalytic results indicate that the catalytic performance of the investigated materials cannot be explained solely by their textural properties. Although CP_KOH_850 exhibited the highest specific surface area and pore volume, no direct correlation between these parameters and geraniol conversion was observed under all reaction conditions. This finding suggests that, besides textural properties, the surface chemistry of the materials, including the concentration of acid sites and the presence of oxygen-containing functional groups, also contributed to the observed catalytic behaviour.

Considering both geraniol conversion and product distribution, the reaction temperatures selected for further catalytic studies were 110 °C for P_POWDER, 95 °C for CP_KOH_750, 100 °C for CP_KOH_800, and 110 °C for CP_KOH_850.

The effect of the amount of catalyst on the catalytic performance of P_POWDER, CP_KOH_750, CP_KOH_800, and CP_KOH_850 in geraniol oxidation was subsequently investigated. Geraniol conversion and product selectivity were used to evaluate the catalytic performance of the investigated materials, and the obtained results are presented in Figure 10.

Comparison of the results presented in Figure 10 shows that the amount of catalyst influenced both geraniol conversion and product selectivity. However, unlike reaction temperature, increasing the amount of catalyst did not result in a systematic increase in geraniol conversion. For P_POWDER, the highest conversion (30 mol%) was obtained using 1 wt% catalyst, whereas further increases in catalyst amount led to a slight decrease in conversion. A similar tendency was observed for CP_KOH_800 and CP_KOH_850, where the highest conversions were achieved at relatively low catalyst amounts (0.5–1 wt%), followed by a gradual decrease with increasing catalyst amount. The most pronounced effect was observed for CP_KOH_750, for which geraniol conversion decreased from 23 to 13 mol% as the catalyst amount increased from 0.5 to 5 wt%.

The amount of catalyst exerted a more noticeable influence on product distribution than on geraniol conversion. For all catalysts, the selectivity toward citral varied only moderately with increasing catalyst amount. For P_POWDER, the selectivity to citral remained within the range of 9–17 mol%, whereas for CP_KOH_750, CP_KOH_800, and CP_KOH_850 the corresponding values ranged from 1–11 mol%, 8–12 mol%, and 6–17 mol%, respectively. Similar behaviour was observed for 2,3-epoxygeraniol and 2,3-epoxycitral, whose selectivities also remained within relatively narrow ranges throughout the investigated catalyst amounts.

More pronounced changes were observed for the fraction of products grouped as “others”. For P_POWDER, the selectivity toward this fraction varied between 56 and 67 mol%. In contrast, for CP_KOH_750 and CP_KOH_850 a marked increase in the selectivity toward “others” was observed at higher catalyst amounts, reaching 99 and 94 mol%, respectively, at 5 wt% catalyst. A similar tendency, although less pronounced, was observed for CP_KOH_800, where the selectivity toward this fraction increased from 64 to 79 mol% as the catalyst amount increased from 0.5 to 5 wt%. These observations indicate that increasing the catalyst amount above a certain level did not improve the formation of the identified oxidation products but favoured the formation of a more complex mixture of oxidation products.

The observed catalytic behaviour suggests that increasing the amount of catalyst above the optimum value does not necessarily improve catalytic performance. Instead, a larger amount of catalyst appears to promote parallel and consecutive reactions, resulting in a broader product distribution. Consequently, the contribution of the “others” fraction increased considerably at higher catalyst amounts. The occurrence of secondary reactions, including condensation and oligomerization processes leading to the formation of higher-molecular-weight products, cannot be excluded under these conditions.

Considering both geraniol conversion and product distribution, the following conditions were selected for the subsequent time-dependent studies: 110 °C with 0.5 wt% catalyst for P_POWDER, 95 °C with 0.7 wt% catalyst for CP_KOH_750, 100 °C with 1 wt% catalyst for CP_KOH_800, and 110 °C with 0.5 wt% catalyst for CP_KOH_850.

The effect of reaction time on the catalytic performance of P_POWDER, CP_KOH_750, CP_KOH_800, and CP_KOH_850 in geraniol oxidation was subsequently investigated, and the results are presented in Figure 11.

Comparison of the results presented in Figure 11 shows that extending the reaction time from 15 to 360 min resulted in a gradual increase in geraniol conversion for all investigated catalysts. For P_POWDER, the conversion increased from 12 to 33 mol%, while for CP_KOH_750, CP_KOH_800, and CP_KOH_850 the corresponding values increased from 12 to 29 mol%, 12 to 29 mol%, and 12 to 37 mol%, respectively. Among the investigated materials, CP_KOH_850 exhibited the highest geraniol conversion after 360 min of reaction.

Reaction time also affected product distribution. The selectivity toward citral generally increased with reaction time for all catalysts. For P_POWDER, CP_KOH_750, and CP_KOH_850, the selectivity to citral increased from approximately 1–2 mol% at the beginning of the reaction to 13 mol% after 360 min. Similar trends were observed for 2,3-epoxygeraniol and 2,3-epoxycitral, whose selectivities gradually increased during the reaction. However, despite the increase in reaction time, the selectivities toward these products remained relatively low, suggesting that they may undergo further transformations under the applied reaction conditions.

The selectivity toward the fraction grouped as “others” decreased with increasing reaction time for all investigated catalysts. For P_POWDER, the selectivity toward this fraction decreased from 98 to 53 mol%, while for CP_KOH_750, CP_KOH_800, and CP_KOH_850 it decreased from 100 to 62 mol%, 100 to 58 mol%, and 99 to 52 mol%, respectively. The decrease in the contribution of this fraction was accompanied by an increase in the selectivities toward the identified oxidation products. This observation suggests that the composition of the “others” fraction evolves throughout the reaction as minor products are formed and transformed. To gain further insight into the composition of this fraction, selected post-reaction mixtures were analysed by GC–MS. The analysis revealed several minor volatile compounds assigned to the fraction grouped as “others”, including farnesol, trans-geranylgeraniol, neryl formate (2,6-octadien-1-ol, 3,7-dimethyl-, formate, (Z)-), and geranylgeranial (Docosa-2,6,10,14,18-pentaen-22-al, 2,6,10,15,18-pentamethyl-, all-trans). These compounds represent only part of the “others” fraction, which may also contain higher-molecular-weight products, including oligomeric species, that are not detectable under the applied GC–MS conditions. Although detected in relatively low amounts, the identified compounds represent chemically relevant minor constituents that may be of potential interest due to the known applications of terpenoid-related compounds in the fragrance, cosmetic and fine chemical industries. Overall, the identification of these compounds provides additional insight into the composition of the “others” fraction and demonstrates that the catalytic oxidation of geraniol generates not only the major oxidation products but also a variety of minor compounds contributing to the complexity of the reaction mixture.

The obtained results demonstrate that reaction time affected both geraniol conversion and product distribution. The highest geraniol conversions together with the highest selectivities toward the identified oxidation products were obtained after 360 min of reaction. Therefore, a reaction time of 360 min was selected for all subsequent catalytic studies.

Overall, the catalytic studies demonstrated that the oxidation of geraniol over the investigated biomass-derived materials was governed by the combined influence of reaction temperature, catalyst amount, reaction time, and catalyst properties. Reaction temperature and catalyst amount primarily affected product distribution, whereas reaction time exerted the strongest influence on geraniol conversion. The absence of a direct correlation between textural properties and catalytic performance indicates that the catalytic behaviour cannot be explained solely by the specific surface area or pore structure of the materials. Instead, surface chemistry and the accessibility of active sites also appear to play important roles in determining both geraniol conversion and product distribution. These findings suggest that the catalytic performance results from the synergistic interplay of several physicochemical properties rather than from any single structural parameter [36]. In addition to pore architecture and surface chemistry, the possible contribution of residual inorganic species cannot be excluded, although confirmation of their role would require further investigation. Among the investigated activated carbons, CP_KOH_850 exhibited the highest catalytic activity under the optimized reaction conditions. These findings provided the basis for the subsequent kinetic analysis of geraniol oxidation over the selected catalysts.

### 3.3. Kinetic Modeling

This work is aimed at exploring the reaction pathways involved in the transformation of geraniol with O_2_, in order to determine suitable kinetic expressions for the rate laws that can mathematically describe all the observed transformations over two heterogeneous catalysts. Kinetic studies available in the literature for this reaction remain limited. Some works have reported different transformation routes of geraniol. For instance, the acetylation of geraniol was investigated by Converti et al. (2002) [37] through biocatalysis catalyzed by lyophilized cells of Aspergillus oryzae, which contain mycelium-bound lipases, using acetic acid in an organic solvent such as n-heptane. Baumstark et al. (2004) [38] studied the epoxidation of geraniol using dimethyldioxirane (DMDO) as the oxidizing agent. The major products were identified as the 2,3- and 6,7- monoepoxides. Although the epoxides were not directly quantified, the disappearance of DMDO was monitored by UV absorbance at 330 nm. Homogeneous kinetic expressions with an overall second-order dependence were employed, and the products were characterized using GC-MS and ^1^H-NMR, enabling the quantification of the product distribution.

In previous work, the use of clinoptilolite, a natural zeolite catalyst [39], was investigated for the chemical transformation of geraniol, where isomerization, dehydration, dimerization, and cyclization pathways were observed. From the literature review, it can be noted that, unlike most studies focused on alternative transformation routes of geraniol, the present work investigates in detail the oxidation of geraniol using molecular oxygen as a green oxidant.

The preliminary kinetic analysis using Equations (1) and (2) revealed that adsorption of all species in the transformation of geraniol had no statistical significance on fitting the experimental data, except for the adsorption of 2,3-epoxygeraniol with a value of (K_B_ = 2.70 × 10^4^ L mol^−1^) over the two heterogeneous catalysts, which can be assumed from our previous study [40]. This value corresponds to the adsorption equilibrium constant of 2,3-epoxygeraniol independently estimated in our earlier kinetic study of geraniol oxidation with molecular oxygen over mironekuton, a natural aluminosilicate mineral catalyst [40]. The consistency of this parameter across two structurally distinct catalytic systems: a biomass-derived activated carbon/powder and a natural clay mineral, supports its physical significance and justifies treating it as a fixed, transferable constant in the present model. This behavior can be rationalized by the bifunctional character of the 2,3-epoxygeraniol molecule, which retains both a hydroxyl group and a proximal, electrophilic epoxide oxygen; this combination enables a bidentate-type interaction with surface oxygen-containing functionalities and acid sites, increasing its residence time on the catalyst surface relative to geraniol, citral, and the structurally heterogeneous “other” products.

Additionally, considering this value as constant allows the remaining kinetic parameters (4 k_i_, 4 Ea_i_) to be adjusted with lower standard errors, as will be shown later in Table 5, indicating that the parameters are statistically reliable. If variation in this parameter (K_B_) is allowed, a similar mathematical fit can be achieved (similar high R^2^ > 99.5%); however, the reliability of the parameters decreases, resulting in very high standard errors, which suggests that the system is over-parameterized.

Table 5 presents the optimized kinetic parameters for the transformation of geraniol using the two tested catalysts. The reaction rate constants estimated at 90 °C for the direct consumption of geraniol showed, for both catalysts, the same trend decreasing order of k_4_ > k_1_ > k_3_ to form products (E), 2,3-epoxygeraniol (B), and citral (D), respectively. This trend was expected due to the order of selectivity toward those products in which the products E are clearly the most selective in the reaction under the tested reaction conditions, followed by 2,3-epoxygeraniol and citral. On the other hand, the formation of 2,3-epoxycitral (C) has the highest reaction rate constant (k_2_ = 1420 and 342 mL g^−1^ min^−1^ for P_POWDER and CP_KOH_850, respectively), which means that the 2,3-epoxygeraniol, which is continuously formed through epoxidation of geraniol with oxygen, is transforming even faster to the formation of 2,3-epoxycitral. Additionally, the formation of 2,3-epoxycitral is favored more over P_POWDER than CP_KOH_850.

Regarding the activation energies, the conversion of geraniol to 2,3-epoxycitral has significant differences in terms of energy barriers to occur because over P_POWDER is required 66.9 kJ mol^−1^, while with CP_KOH_850 is required 159 kJ mol^−1^. The activation energy for the formation of 2,3-epoxygeraniol is higher over CP_KOH_850 than P_POWDER, while the activation energies for the formation of citral and products E are quite similar (74–77 kJ mol^−1^ and 39.9–43.1 kJ mol^−1^, respectively).

**Figure 12:**
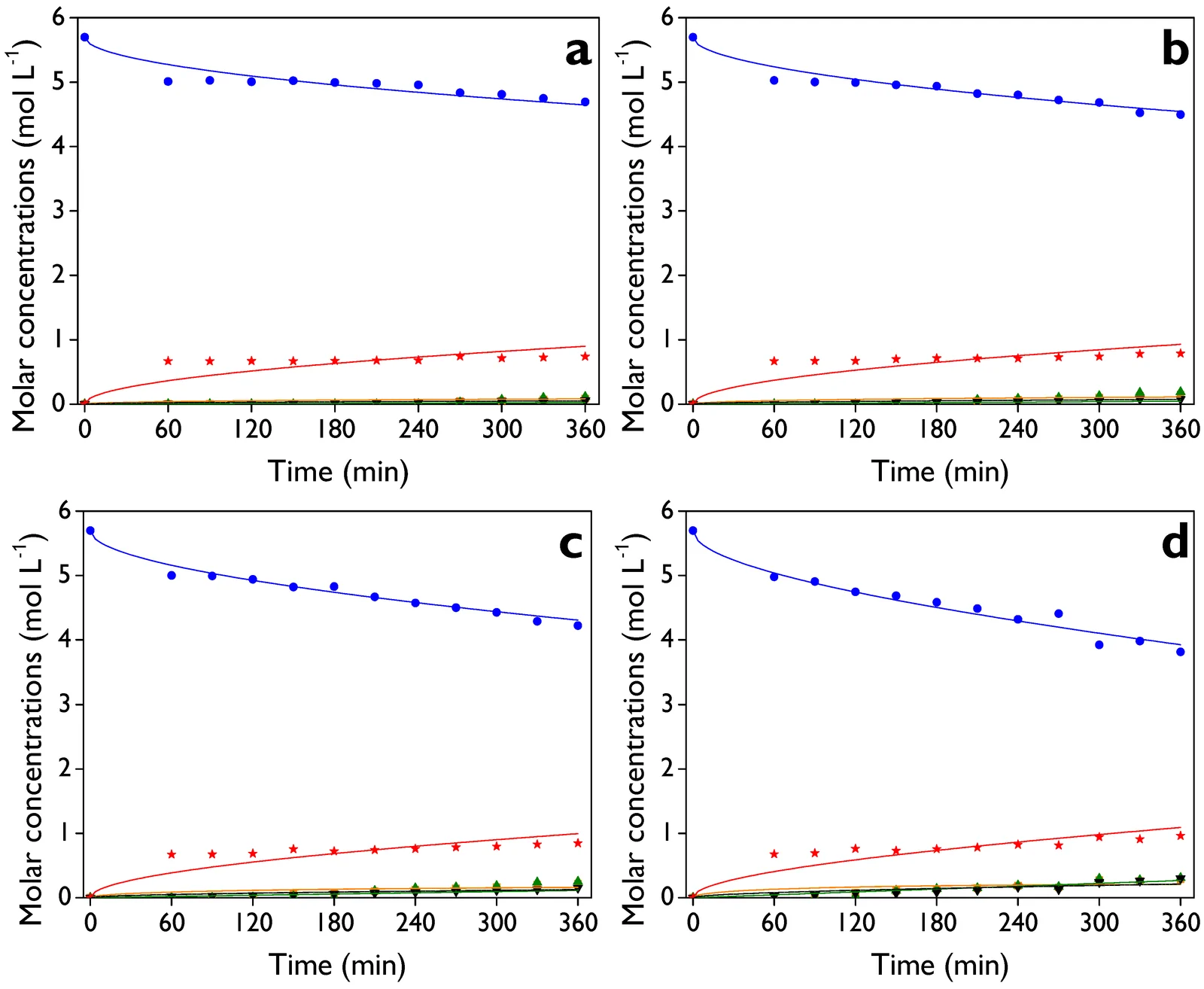
Concentration profiles of the species (C_A_ (—, ●), C_B_ (—, ■), C_C_ (—, ▲), C_D_ (—, ▼), C_E_ (—, ★)) involved in the transformation of geraniol over P_POWDER, with experimental values (symbols) and modeled values (solid lines). Reaction conditions: 20 g of geraniol, 100 mg of catalyst, free solvent. (**a**) 70 °C, (**b**) 80 °C, (**c**) 95 °C, and (**d**) 110 °C.

**Figure 13:**
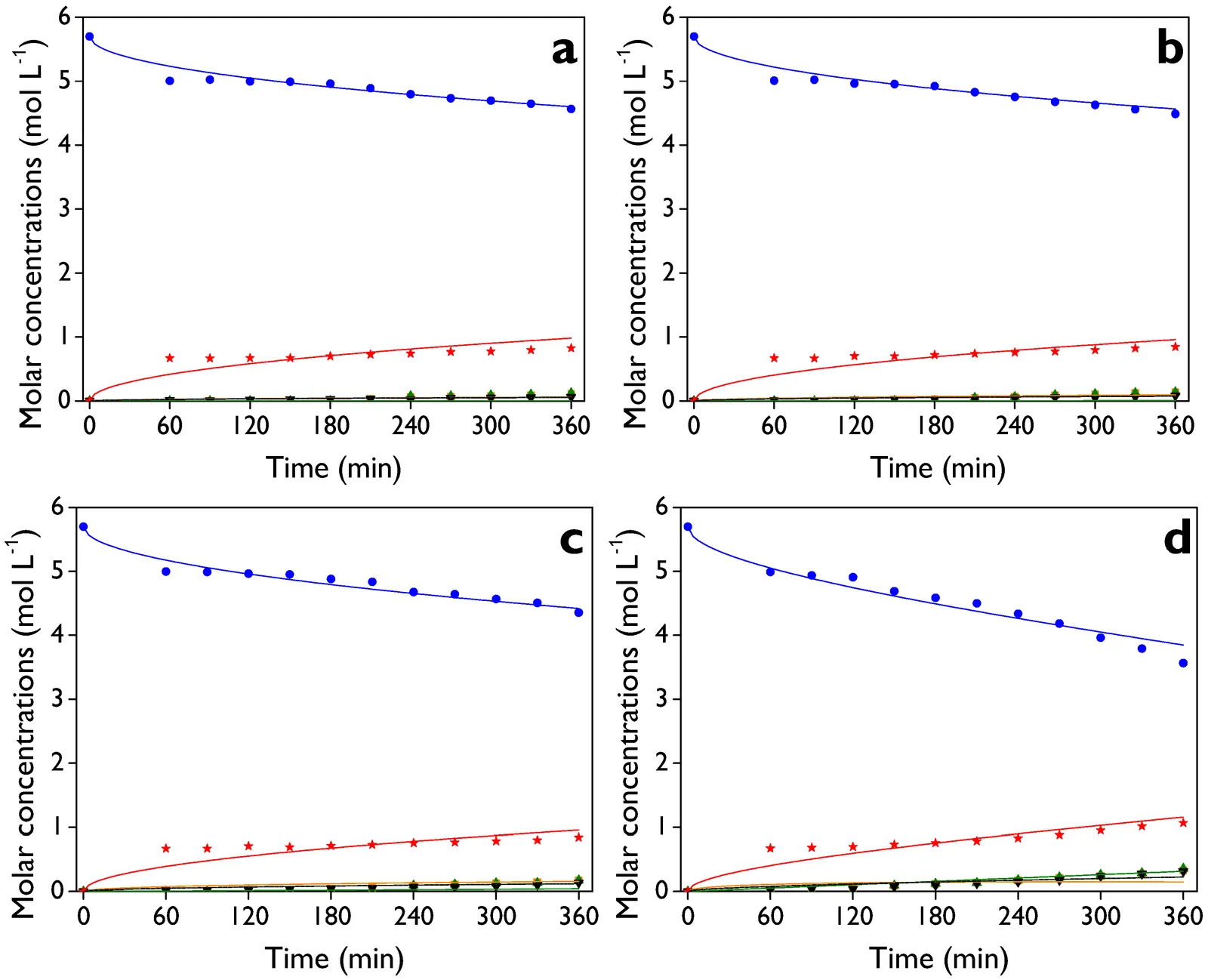
Concentration profiles of the species (C_A_ (—, ●), C_B_ (—, ■), C_C_ (—, ▲), C_D_ (—, ▼), C_E_ (—, ★)) involved in the transformation of geraniol over CP_KOH_850 with experimental values (symbols) and modeled values (solid lines). Reaction conditions: 20 g of geraniol, 100 mg of catalyst, free solvent. (**a**) 70 °C, (**b**) 80 °C, (**c**) 95 °C, and (**d**) 110 °C.

The comparison between the experimental concentration profiles and those calculated using the kinetic model is illustrated in Figure 12 and Figure 13 for P_POWDER and CP_KOH_850, respectively. These plots show that the proposed kinetics model successfully reproduces the behavior of the experimental data, yielding a high R^2^ value (>99.78%) and exhibiting similar trends between the modeled and experimental curves. Furthermore, the relatively low standard errors demonstrate the reliability of the obtained kinetic parameters. On the other hand, Table 6 presents the correlation matrices obtained for the kinetic parameters associated with the two heterogeneous catalysts. These correlation coefficients reveal how strongly each parameter is linearly related to the others, where values close to ±1 indicates strong relationships. For both catalysts, a strong positive correlation was observed between k_1_ and k_4_ (r = 0.952 and 0.980 for P_POWDER and CP_KOH_850, respectively) and Ea_1_ and Ea_4_ (r = 0.968 and 0.988 for P_POWDER and CP_KOH_850, respectively).

### 3.4. Comparison of the Obtained Results with Literature Reports

To place the obtained results in a broader scientific context, the catalytic performance of the prepared activated carbons was compared with selected literature reports on heterogeneous catalysts used for geraniol oxidation. A summary of the comparison is presented in Table 7.

Among the literature reports summarized in Table 7, Dapurkar et al. [41] investigated the selective oxidation of geraniol over a CrMCM-41 catalyst using molecular oxygen under supercritical CO_2_ conditions. The catalyst achieved a geraniol conversion of 53 mol% with an excellent total citral selectivity of 98 mol%, demonstrating the high efficiency of the CrMCM-41 catalyst for the selective oxidation of geraniol to citral. However, the process required a synthetically prepared Cr-containing catalyst and operation under high-pressure supercritical CO_2_ (14 MPa). Cubillos et al. [42] reported the oxidation of geraniol over hydrogen peroxide-treated niobium oxide (Nb_2_O_5_/H_2_O_2_), obtaining a lower conversion (17 mol%) but an outstanding selectivity towards 2,3-epoxygeraniol (80 mol%) under relatively mild conditions. In turn, da Silva et al. [43] achieved the highest geraniol conversion (>90 mol%) using vanadium-substituted phosphomolybdic acids. Their excellent catalytic performance resulted from the combined redox and acidic properties of the catalyst but required hydrogen peroxide, acetonitrile, and a synthetically prepared V- and Mo-containing catalyst.

In contrast to these synthetic metal-containing catalysts, our previous studies focused on naturally occurring mineral catalysts operating under solvent-free conditions with molecular oxygen as the oxidizing agent. Vermiculite [1] exhibited moderate catalytic activity, reaching a geraniol conversion of 22 mol% while producing several oxidation products, including citral, 2,3-epoxygeraniol, and 2,3-epoxycitral. A subsequent study employing mironekuton [40] demonstrated that acid modification markedly enhanced catalytic performance. Under the optimized reaction conditions summarized in Table 7, geraniol conversion increased from 42 mol% for the pristine material to 99 mol% for the acid-modified catalyst, accompanied by a substantial shift in product distribution towards thunbergol formation.

The present study extends this approach by investigating biomass-derived activated carbons prepared from *Pelargonium zonale* as heterogeneous catalysts for geraniol oxidation. Under the optimized reaction conditions (110 °C, 0.5 wt% catalyst, 6 h), the highest geraniol conversion reached 37 mol%. Although this value was lower than those reported for the most active synthetic catalysts, the activated carbons effectively promoted geraniol oxidation under environmentally friendly solvent-free conditions using molecular oxygen as the oxidant. The reaction produced a relatively balanced distribution of oxidation products, with 2,3-epoxycitral (17 mol%), 2,3-epoxygeraniol (16 mol%), and citral (15 mol%) being the major products, indicating that the biomass-derived carbon catalysts promoted several parallel oxidation pathways rather than favouring a single dominant reaction.

Unlike the synthetic catalysts summarized above, the activated carbons investigated in this study were produced from post-consumer *Pelargonium zonale* biomass, providing an effective route for the valorization of plant waste into functional heterogeneous catalysts. Furthermore, the systematic comparison of activated carbons differing in textural properties and surface chemistry provided valuable insight into the relationship between catalyst physicochemical characteristics and catalytic performance.

Overall, the comparison presented in Table 7 demonstrates that catalytic performance depends strongly on both catalyst composition and reaction conditions. Synthetic metal-containing catalysts generally provide the highest geraniol conversions and product selectivities; however, they often require complex synthesis procedures, hydrogen peroxide as the oxidizing agent, organic solvents, or high-pressure reaction systems. In contrast, naturally occurring mineral catalysts and biomass-derived activated carbons constitute more sustainable alternatives, operating under solvent-free conditions with molecular oxygen. Although the activated carbons developed in the present study exhibited lower activity than the best synthetic catalysts, they successfully combined renewable biomass valorization with environmentally benign catalytic oxidation and provided new insight into the influence of textural and surface properties on the catalytic behaviour of biomass-derived activated carbons during geraniol oxidation.

## 4. Conclusions

The present study demonstrated that the aerial parts of *Pelargonium zonale*, treated as post-consumer plant biomass, can be successfully converted into activated carbon materials by chemical activation with KOH. Increasing the carbonization temperature promoted pore development, resulting in activated carbons with high specific surface areas and well-developed porous structures. Although the textural properties of the obtained materials differed considerably, their surface chemistry remained relatively similar, indicating that the activation temperature primarily affected the porous structure of the carbons.

The catalytic studies confirmed that all investigated materials were active in the oxidation of geraniol; however, their catalytic performance depended on both the reaction conditions and the physicochemical properties of the catalysts. Optimization of the reaction parameters demonstrated that reaction temperature, catalyst amount, and reaction time influenced both geraniol conversion and product distribution. No direct correlation between the textural properties of the activated carbons and their catalytic performance was observed. The acid site concentrations determined by titration were very similar for all activated carbons, which is consistent with their comparable selectivities. However, the higher geraniol conversion observed for CP_KOH_850 indicates that total acidity alone does not determine the catalytic performance. Likewise, only minor differences in the FTIR spectra were observed after activation. These results suggest that the catalytic activity arises from the combined influence of several physicochemical properties, including the accessibility of active sites within the porous structure and, potentially, the presence of residual inorganic species. Consequently, no single physicochemical parameter can satisfactorily explain the catalytic behaviour of the investigated materials. Among the investigated activated carbons, CP_KOH_850 exhibited the highest geraniol conversion under the optimized reaction conditions.

Kinetic analysis demonstrated that geraniol oxidation proceeds through a complex reaction network involving several parallel and consecutive pathways leading to the formation of citral, 2,3-epoxygeraniol, 2,3-epoxycitral, and other oxidation products. The proposed kinetic model successfully described the experimental results and provided additional insight into the reaction network and product formation over the investigated biomass-derived catalysts.

Overall, the obtained results demonstrate that waste biomass from *Pelargonium zonale* represents a promising renewable precursor for the preparation of functional activated carbon materials. The combination of comprehensive physicochemical characterization, catalytic evaluation, and kinetic modelling provides valuable insight into the design of biomass-derived carbon catalysts and supports the sustainable valorization of plant waste into value-added functional materials.

## Figures and Tables

**Table 1:** Production rate for each species j.

Specie	Production Rate (R_j_)
Geraniol (A)	$-r_{1}-r_{3}-r_{4}$
2,3-Epoxygeraniol (B)	$r_{1}-r_{2}$
2,3-Epoxycitral (C)	$r_{2}$
Citral (D)	$r_{3}$
Other products (E)	$r_{4}$

**Table 2:** Textural characteristics of CP_KOH_750, CP_KOH_800, CP_KOH_850.

Sample Name	SSA	V_tot_	V_micro_	V_micro_/V_tot_	D_av_
	(m^2^g^−1^)	(cm^3^g^−1^)	(cm^3^g^−1^)	(%)	(nm)
CP_KOH_750	1312	0.587	0.442	75	1.79
CP_KOH_800	1417	0.646	0.488	76	1.82
CP_KOH_850	1578	0.751	0.533	71	1.90

SSA: Specific surface area.

**Table 3:** Elemental analysis of P_POWDER and of activated carbons prepared from *Pelargonium zonale* and activated by KOH (wt%).

Element	P_POWDER	CP_KOH_750	CP_KOH_800	CP_KOH_850
Ca	6.30	2.60	3.73	3.49
K	4.44	0.36	0.27	0.31
Cl	1.66	0.18	0.089	0.17
P	0.40	0.02	0.02	0.03
Si	0.33	1.97	0.94	1.54

**Table 4:** The comparison of the acid site concentration in pristine P_POWDER, CP_KOH_750, CP_KOH_800, and CP_KOH_850.

Catalyst Type	Acid Site Concentration [mmol/g]
P_POWDER	2.37
CP_KOH_750	0.96
CP_KOH_800	0.80
CP_KOH_850	0.89

**Table 5:** Optimized kinetic parameters for the kinetic model.

Parameter	Units	P_POWDER		CP_KOH_850	
		Value	Standard Error (%)	Value	Standard Error (%)
k_1_	mL g^−1^ min^−1^	66.2	17.8	38.9	22.8
k_2_	mL g^−1^ min^−1^	1420	22.5	342.0	56.7
k_3_	mL g^−1^ min^−1^	30.6	16.0	25.5	15.5
k_4_	mL g^−1^ min^−1^	284	9.0	233.0	11.2
Ea_1_	kJ mol^−1^	75.6	16.1	94.0	16.5
Ea_2_	kJ mol^−1^	66.9	19.8	159.0	19.5
Ea_3_	kJ mol^−1^	74.0	14.8	77.0	13.7
Ea_4_	kJ mol^−1^	39.9	15.4	43.1	18.3
^a^ R^2^	-	99.78	-	99.80	-

k_i_ values were estimated at 90 °C. ^a^ R^2^ refers to the determination coefficient of the fitting. K_B_ = 2.70 × 10^4^ L mol^−1^ [40].

**Table 6:** Correlation matrices of the parameters for both catalysts.

**Catalyst: P_POWDER**								
	**k_1_**	**k_2_**	**k_3_**	**k_4_**	**E_a1_**	**E_a2_**	**E_a3_**	**E_a4_**
**k_1_**	1.000							
**k_2_**	0.354	1.000						
**k_3_**	0.172	−0.200	1.000					
**k_4_**	0.952	0.103	0.172	1.000				
**E_a1_**	−0.683	−0.275	−0.098	−0.641	1.000			
**E_a2_**	−0.303	−0.870	0.173	−0.118	0.360	1.000		
**E_a3_**	−0.100	0.143	−0.699	−0.097	0.196	−0.205	1.000	
**E_a4_**	−0.653	−0.171	−0.096	−0.626	0.968	0.193	0.191	1.000
**Catalyst: CP_KOH_850**								
	**k_1_**	**k_2_**	**k_3_**	**k_4_**	**E_a1_**	**E_a2_**	**E_a3_**	**E_a4_**
**k_1_**	1.000							
**k_2_**	0.180	1.000						
**k_3_**	0.407	−0.124	1.000					
**k_4_**	0.980	0.042	0.384	1.000				
**E_a1_**	−0.779	−0.225	−0.310	−0.740	1.000			
**E_a2_**	−0.169	−0.987	0.123	−0.042	0.264	1.000		
**E_a3_**	−0.333	−0.039	−0.686	−0.288	0.446	0.027	1.000	
**E_a4_**	−0.759	−0.273	−0.275	−0.705	0.988	0.299	0.420	1.000

**Table 7:** Comparison of catalytic systems reported for geraniol oxidation under different reaction conditions.

Ref.	Catalyst	Reaction Conditions *	Conv. (mol%) *	Main Products (Sel., mol%) *	Key Features
[41]	CrMCM-41	O_2_/scCO_2_, 80 °C, 6 h	53	Citral (98)	High citral selectivity; recyclable catalyst
[42]	Nb_2_O_5_	H_2_O_2_/N_2_, RT, 4 h	17	Epoxygeraniol (80); citral (14)	Mild conditions; recyclable catalyst
[43]	H_4_PMo_11_VO_40_	H_2_O_2_/CH_3_CN, 60 °C, 8 h	>90	Epoxygeraniol (80–85)	High conversion and epoxygeraniol selectivity
[1]	Vermiculite	O_2_, solvent-free, 90 °C, 4 h	22	2,3-Epoxygeraniol (10); citral (9); 2,3-epoxycitral (6)	Natural mineral catalyst
[40]	Mironekuton (MIR)	O_2_, solvent-free, 90 °C, 5 wt%, 3 h	42	Thunbergol (20); 6,11-dimethyldodeca-2,6,10-trien-1-ol (18); Linalool (6); Citral (5);	Natural catalyst
[40]	Acid-modified mironekuton (MIR_MOD)	O_2_, solvent-free, 90 °C, 5 wt%, 3 h	99	Thunbergol (35); 6,11-dimethyldodeca-2,6,10-trien-1-ol (9); Ocimenes (5); Linalool (4)	Improved activity after acid treatment
**This work**	Biomass-derived activated carbon (CP_KOH_850)	O_2_, solvent-free, 110 °C, 0.5 wt%, 6 h	37	2,3-Epoxycitral (17); 2,3-epoxygeraniol (16); citral (15)	Biomass-derived catalyst

* Reaction conditions correspond to the optimum conditions reported in each study. Conv.—geraniol conversion; Sel.—product selectivity; RT—room temperature; scCO_2_—supercritical carbon dioxide; CH_3_CN—acetonitrile.

## Data Availability

The original contributions presented in this study are included in the article. Further inquiries can be directed to the corresponding authors.

## Supplementary Materials

The following supporting information can be downloaded at: https://www.mdpi.com/article/10.3390/ma19163468/s1, Figure S1: Elemental steps proposed for the reaction pathway; Equations (S1)–(S25).
