# Supplementary material for: Pelargonium zonale as a Source of Active Catalysts for the Oxidation of Geraniol with Oxygen
Source: Materials (Basel). 2026 Aug 17;19(16):3468. doi: 10.3390/ma19163468 (PMC13514215; doi:10.3390/ma19163468)
Supplement: Supplementary file 1 [file materials-19-03468-s001.zip › materials-4444441-supplementary.pdf]

SUPPORTING INFORMATION

1. Kinetic modeling

The reaction rate laws for the four reactions of the reaction network (Figure 2 in the article) are obtained using heterogeneous kinetic models as deduced from the steps illustrated in Figure S1.

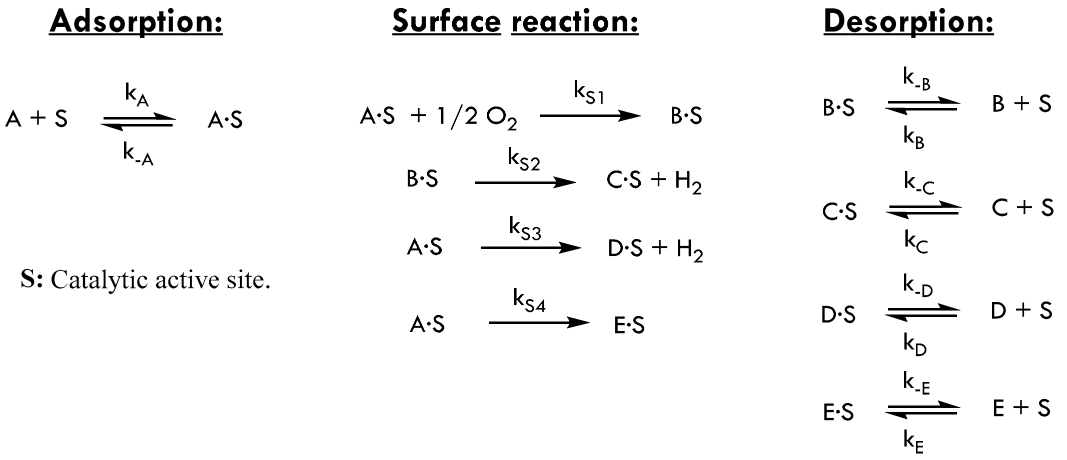

Figure S1. Elemental steps proposed for the reaction pathway.

S: Surface site on catalyst.
C_i: Concentration of specie i in the bulk of the solution.
C_v: Concentration of available surface site on the catalyst surface.
C_{is}: Concentration of specie i adsorbed at the surface site.
C_t: Total number of sites available on the catalyst surface.
k_i: Reaction rate constant of direct reaction i.
k_{-i}: Reaction rate constant of reverse reaction i.
K_i: Equilibrium constant for step i.
P_{O_2}: The partial pressure of oxygen is considered constant because a pure oxygen flow is used.

$$r_{A,ads} = k_A\left[C_A C_V - \frac{C_{A\cdot S}}{K_A}\right] \tag{S1}$$

$$r_1 = k_{s1} C_{A\cdot S} P_{O_2}^{1/2} = k'_{s1} C_{A\cdot S} \tag{S2}$$

$$r_2 = k_{s2} C_{B\cdot S} \tag{S3}$$

$$r_3 = k_{s3} C_{A\cdot S} \tag{S4}$$

$$r_4 = k_{s4} C_{A\cdot S} \tag{S5}$$

$$r_{B,des} = k_{-B}\left[C_{B\cdot S} - \frac{C_B C_V}{K_{B,d}}\right] \tag{S6}$$

$$r_{C,des} = k_{-C}\left[C_{C\cdot S} - \frac{C_C C_V}{K_{C,d}}\right] \tag{S7}$$

$$r_{D,des} = k_{-D}\left[C_{D\cdot S} - \frac{C_D C_V}{K_{D,d}}\right] \tag{S8}$$

$$r_{E,des} = k_{-E}\left[C_{E\cdot S} - \frac{C_E C_V}{K_{E,d}}\right] \tag{S9}$$

The Eqs. (S1), and (S6)-(S9) are solved to find the concentrations of adsorbed species (C_{is}) when these are equal to zero, assuming the surface reactions (Eqs. (S2)-(S5)) as the rate-limiting steps.

$$C_{A\cdot S} = K_A C_A C_V \tag{S10}$$

$$C_{B\cdot S} = \frac{C_B C_V}{K_{B,d}} = K_B C_B C_V \tag{S11}$$

$$C_{C\cdot S} = \frac{C_C C_V}{K_{C,d}} = K_C C_C C_V \tag{S12}$$

$$C_{D\cdot S} = \frac{C_D C_V}{K_{D,d}} = K_D C_D C_V \tag{S13}$$

$$C_{E\cdot S} = \frac{C_E C_V}{K_{E,d}} = K_E C_E C_V \tag{S14}$$

The balance of sites is defined by Eq. (S15):

$$C_t = C_V + C_{A\cdot S} + C_{B\cdot S} + C_{C\cdot S} + C_{D\cdot S} + C_{E\cdot S} \tag{S15}$$

By replacing Eqs. (S10) – (S14) in Eq. (S15) and simplifying, Eq. (S16) is obtained:

$$C_t = C_V + K_A C_A C_V + K_B C_B C_V + K_C C_C C_V + K_D C_D C_V + K_E C_E C_V$$

$$C_V = \frac{C_t}{1 + K_A C_A + K_B C_B + K_C C_C + K_D C_D + K_E C_E} \tag{S16}$$

Replacing Eq. (S16) in Eqs. (S10) - (S14):

$$C_{A\cdot S} = \frac{K_A C_A C_t}{1 + K_A C_A + K_B C_B + K_C C_C + K_D C_D + K_E C_E} \tag{S17}$$

$$C_{B\cdot S} = \frac{K_B C_B C_t}{1 + K_A C_A + K_B C_B + K_C C_C + K_D C_D + K_E C_E} \tag{S18}$$

$$C_{C\cdot S} = \frac{K_C C_C C_t}{1 + K_A C_A + K_B C_B + K_C C_C + K_D C_D + K_E C_E} \tag{S19}$$

$$C_{D\cdot S} = \frac{K_D C_D C_t}{1 + K_A C_A + K_B C_B + K_C C_C + K_D C_D + K_E C_E} \tag{S20}$$

$$C_{E\cdot S} = \frac{K_E C_E C_t}{1 + K_A C_A + K_B C_B + K_C C_C + K_D C_D + K_E C_E} \tag{S21}$$

The final expressions for the reaction rates of r_1-r_4 are shown below, where $k_i = k_{si} K_i C_t$:

$$r_1 = \frac{k_1 C_A}{1 + K_A C_A + K_B C_B + K_C C_C + K_D C_D + K_E C_E} \tag{S22}$$

$$r_2 = \frac{k_2 C_B}{1 + K_A C_A + K_B C_B + K_C C_C + K_D C_D + K_E C_E} \tag{S23}$$

$$r_3 = \frac{k_3 C_A}{1 + K_A C_A + K_B C_B + K_C C_C + K_D C_D + K_E C_E} \tag{S24}$$

$$r_4 = \frac{k_4 C_A}{1 + K_A C_A + K_B C_B + K_C C_C + K_D C_D + K_E C_E} \tag{S25}$$
